# Environmentally Induced Epigenetic Transgenerational Inheritance of Altered Sertoli Cell Transcriptome and Epigenome: Molecular Etiology of Male Infertility

**DOI:** 10.1371/journal.pone.0059922

**Published:** 2013-03-28

**Authors:** Carlos Guerrero-Bosagna, Marina Savenkova, Md. Muksitul Haque, Eric Nilsson, Michael K. Skinner

**Affiliations:** Center for Reproductive Biology, School of Biological Sciences, Washington State University, Pullman, Washington, United States of America; Massachusetts General Hospital, United States of America

## Abstract

Environmental toxicants have been shown to induce the epigenetic transgenerational inheritance of adult onset disease, including testis disease and male infertility. The current study was designed to determine the impact of an altered sperm epigenome on the subsequent development of an adult somatic cell (Sertoli cell) that influences the onset of a specific disease (male infertility). A gestating female rat (F0 generation) was exposed to the agriculture fungicide vinclozolin during gonadal sex determination and then the subsequent F3 generation progeny used for the isolation of Sertoli cells and assessment of testis disease. As previously observed, enhanced spermatogenic cell apoptosis was observed. The Sertoli cells provide the physical and nutritional support for the spermatogenic cells. Over 400 genes were differentially expressed in the F3 generation control versus vinclozolin lineage Sertoli cells. A number of specific cellular pathways were identified to be transgenerationally altered. One of the key metabolic processes affected was pyruvate/lactate production that is directly linked to spermatogenic cell viability. The Sertoli cell epigenome was also altered with over 100 promoter differential DNA methylation regions (DMR) modified. The genomic features and overlap with the sperm DMR were investigated. Observations demonstrate that the transgenerational sperm epigenetic alterations subsequently alters the development of a specific somatic cell (Sertoli cell) epigenome and transcriptome that correlates with adult onset disease (male infertility). The environmentally induced epigenetic transgenerational inheritance of testis disease appears to be a component of the molecular etiology of male infertility.

## Introduction

Environmentally induced epigenetic transgenerational inheritance of adult onset disease [1] can be promoted by factors such as toxicants [2], [3] or nutrition [4], [5], [6], [7]. Environmental chemicals shown to promote transgenerational disease include the fungicide vinclozolin [2], [8], plastics (bisphenol A (BPA) and phthalates DEHP and DBP) [3], [9], [10], pesticides (methoxychlor and permethrin) [2], [3], dioxin [3], [11], and hydrocarbons [3]. A number of transgenerational diseases/abnormalities have been shown to be induced such as testis disease [2], [9], [12], prostate disease [13], [14], kidney disease [7], [14], ovarian disease [3], [15], reproductive tract abnormalities [14], brain and behavior abnormalities [10], [16], [17], and immune abnormalities [14]. Environmentally induced transgenerational phenomena have been observed in plants [18], flies [19], [20], worms [21], [22], rodents [2], [11], and humans [23], [24]. The current study was designed to investigate the actions of a specific toxicant (vinclozolin) to promote a transgenerational alteration in a somatic cell (Sertoli) that correlates to the induction of disease in the tissue (testis).

Transgenerational phenotypes involve the germline transmission of epigenetic alterations (e.g. DNA methylation) in the absence of any direct environmental exposures [1], [25]. Environmental exposures during fetal gonadal sex determination modifies the epigenetic (DNA methylation) programming of the germline to induce permanently programmed differential DNA methylation regions (DMR) that then transmit an altered epigenome to the subsequent generation [1], [26]. Normal primordial germ cell development in the gonad requires the erasure and re-methylation of the DNA to promote the development of a male (sperm) versus female (egg) germline [26], [27], [28]. The somatic cells and tissues derived from this epigenetically altered germline will promote all somatic cells to develop a modified epigenome and transcriptome [29]. Each tissue will develop an organ specific transgenerational transcriptome [29], [30] that is associated with the disease/abnormality of the tissue [29]. An example provided is the vinclozolin induced transgenerational ovarian disease that correlates with an altered granulosa cell epigenome and transcriptome associated with the development of polycystic ovarian disease [15]. This provides insights into the molecular etiology of disease development within the tissue.

The testis is the site of spermatogenesis that occurs within seminiferous tubules composed of Sertoli cells and an adjacent basal layer of mesenchymal peritubular cells [31], [32]. The interstitial tissue between tubules is composed of Leydig cells, the site of testosterone production, and testicular macrophages [33], [34]. All these somatic cells cooperate in testicular function to support germ cell development and production [31], [32]. The most critical cell for the support of the developing spermatogenic cells is the Sertoli cell that provides the physical support, formation of the blood testis barrier, and nutritional factors needed for spermatogenesis [32]. The Sertoli cells synthesize a number of transport binding proteins (e.g. transferrin) to carry essential factors (e.g. iron) to the developing germ cells [32]. In addition, the Sertoli cells produce pyruvate/lactate that is used as the primary energy metabolite by the germ cells that are sequestered within the blood testis barrier and not able to acquire glucose [35], [36].

Testis disease is primarily associated with abnormal spermatogenesis and reduced sperm counts leading to male infertility [37], [38]. This can be the result of developmental defects such as cryptorchidism or hypospadias [39]. A number of studies have suggested environmental exposures promote testis abnormalities and disease [3], [12], [40]. This includes endocrine disruptors and environmental chemicals. A number of histopathologies have been associated with testis disease and termed testicular dysgenesis syndrome [38], [39], [40]. One of the primary abnormalities is increased spermatogenic cell apoptosis resulting in reduced sperm numbers [41], [42]. The male infertility associated with testis disease has increased dramatically over the past decades and now affects over 10% of the human male population [43]. Although abnormal endocrinology and environmental factors have been associated with testis disease [44], the specific molecular etiologies remain to be elucidated.

Previous studies have demonstrated that exposure of a gestating female to the agriculturally used fungicide vinclozolin [2] during gonadal sex determination promotes the epigenetic transgenerational inheritance of adult onset testis disease [2], [12], [14], [45]. In the F3 generation 90% of the males had a transgenerational spermatogenic cell defect of enhanced apoptosis and reduced sperm count and motility [2], [12]. The objective of the current study was to utilize this model to elucidate components of the molecular etiology of male infertility. Since the Sertoli cell is the primary somatic cell that supports the development of the spermatogenic cells, the role of the Sertoli cell in mediating the transgenerational testis disease is investigated. Observations demonstrate that the vinclozolin induced epigenetic transgenerational inheritance of spermatogenic cell defects and testis disease is associated with a transgenerational alteration in the Sertoli cell epigenome and transcriptome.

## Results

### Transgenerational Spermatogenic Cell Abnormality

**Figure 1:**
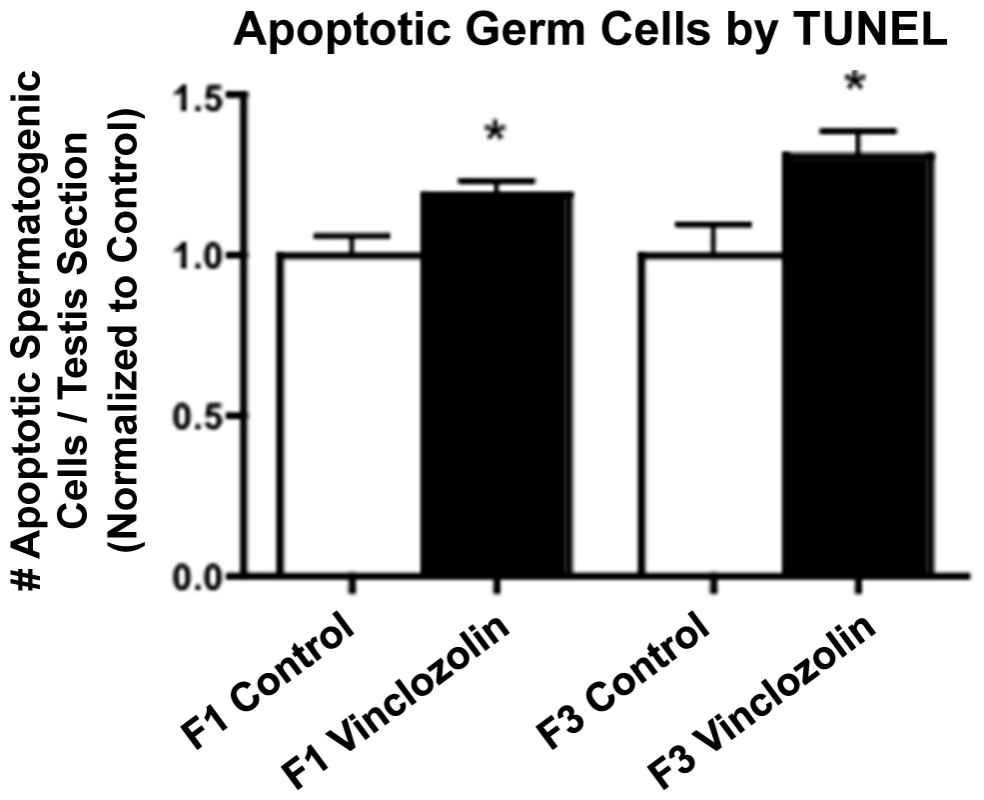
Spermatogenic cell apoptosis in 1-year-old males. Relative apoptotic spermatogenic cell number per testis section is presented. F1 and F3 generation control and vinclozolin lineage testis were examined with the mean ± SEM presented and asterisks (*) indicating a statistically significant difference (p<0.05).

The experimental design involved the exposure of a gestating female (F0 generation) Sprague-Dawley rat to vinclozolin (100 mg/kg/day) during days 8–14 (E8–14) of fetal development [2], [12]. The F1 generation progeny were bred to obtain the F2 generation and the F2 bred to obtain the F3 generation [2], [12]. No sibling or cousin breeding was used in order to avoid any inbreeding artifacts. Corresponding control (vehicle DMSO) lineage F1, F2 and F3 generations were obtained to compare to the vinclozolin lineage males. Some of the direct exposure F1 generation and transgenerational F3 generation control and vinclozolin lineage males were aged to 1 year and testes collected to examine the number of apoptotic spermatogenic cells as previously described [2], [12]. The F1 and F3 generation vinclozolin lineage males had an increased number of apoptotic spermatogenic cells (p<0.05), Figure 1. As previously described [2], [12], the vinclozolin lineage animals had spermatogenic cell defects that correlated to testis disease and male infertility [14].

Another set of F3 generation males from the control and vinclozolin lineages were aged to 20 days of postnatal development and testes collected to isolate purified populations of Sertoli cells. At this age of development no spermatogenic cell apoptosis has been observed, so avoid disease artifacts, and the highest purity cell preparations are obtained [2], [12]. The isolated Sertoli cell populations from three different groups of animals and cell isolates were used to obtain Sertoli cell RNA and DNA from F3 generation control and vinclozolin lineages.

### Sertoli Cell Transgenerational Transcriptome

**Figure 2:**
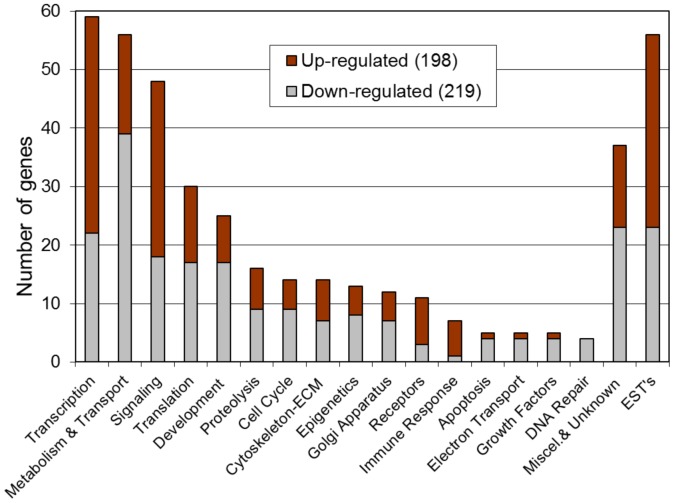
Gene functional categories in F3 generation vinclozolin lineage Sertoli cells differentially expressed gene (417 genes). The number of genes associated with the different functional categories are presented for up-regulated (black bar) or down-regulated (gray bar).

The Sertoli cell RNA was used in a microarray analysis and the quality of the RNA and array monitored. The comparison of the microarrays demonstrated nearly identical profiles on the arrays, Supplemental Figure S1. No batch effects were observed due to cell or RNA isolation dates. The differential gene expression between the control and vinclozolin lineage F3 generation Sertoli cell arrays was determined with a statistical difference (p<0.05), fold change (>1.2 fold) and mean difference (>10) as cut off limits, described in the Methods. The comparison identified 417 differentially expressed genes in the F3 generation Sertoli cell control versus vinclozolin comparison, Supplemental Table S1. The differentially expressed genes involved 198 genes with up-regulation and 219 genes with down-regulation, Figure 2. The functional gene categories of transcription, metabolism, signaling and development were predominant.

**Figure 3:**
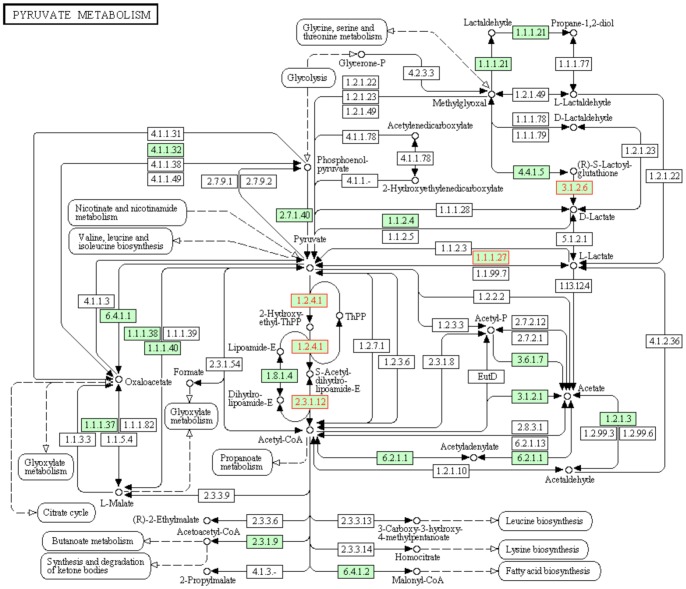
Pyruvate and lactate metabolic pathway with colored genes having significantly different altered expression in the F3 generation vinclozolin lineage Sertoli cells.

A cellular signaling and process pathway analysis was performed as previously described [46]. Analysis of the 417 differentially expressed genes identified 22 pathways with variable impact factors and statistically significant over-representation, Table 1. One of the top pathways most relevant to the Sertoli cell was the pyruvate/lactate metabolism pathway, Figure 3. A number of the key enzymes in pyruvate and lactate metabolism were modified that influence pyruvate and lactate production by the Sertoli cell. These are subsequently a required energy source for the spermatogenic cells. The key enzymes that are all significantly down regulated (>2 fold) were hydroxyacyl-glutathione hydrolase (*Hagh*), pyruvate dehydrogenase beta (*Pdhb*), lactate dehydrogenase A like bb (*Ldnalbb*), and dihydrollipoxamideS-acetyltransferase (*Dlat*), Figure 3. Other prominent pathways affected, Table 1, were the proteasome, nucleotide excision repair, RNA transport, p53 signaling, and mTOR signaling (Supplemental Figure S2). The pathway class most affected was Genetic Information Processing, Table 1.

**Table 1:** Associated Functional Pathway Categories.

Functional Pathway Category	Pathway	Altered Genesin Pathway	Total Genesin Pathway	Impact Factor
	Oxidative phosphorylation	13	207	NA
**Metabolism**	Pyruvate metabolism	4	73	NA
	Glycerophospholipid metabolism	4	82	NA
	Proteasome	5	51	7.5
	Nucleotide excision repair	4	43	5.8
**Genetic Information Processing**	RNA transport	10	134	NA
	Protein processing in endoplasmic reticulum	7	137	NA
	Spliceosome	6	120	NA
	mTOR signaling pathway	4	54	5.9
	Phosphatidylinositol signaling system	4	70	18.9
**Signal Transduction**	ErbB signaling pathway	4	83	3.8
	Jak-STAT signaling pathway	5	136	4.5
	MAPK signaling pathway	7	253	1.8
	p53 signaling pathway	5	67	4.8
**Cellular Processes**	Cell cycle	8	117	6.1
	Oocyte meiosis	5	109	NA
	Focal adhesion	4	187	3.2
**Organismal Systems/Immune System**	Fc epsilon RI signaling pathway	4	70	4.2
	Chemokine signaling pathway	4	189	NA
	Progesterone-mediated oocyte maturation	5	88	NA
**Organismal Systems/Endocrine System**	GnRH signaling pathway	4	91	2.8
	Insulin signaling pathway	4	130	2.6

**Figure 4:**
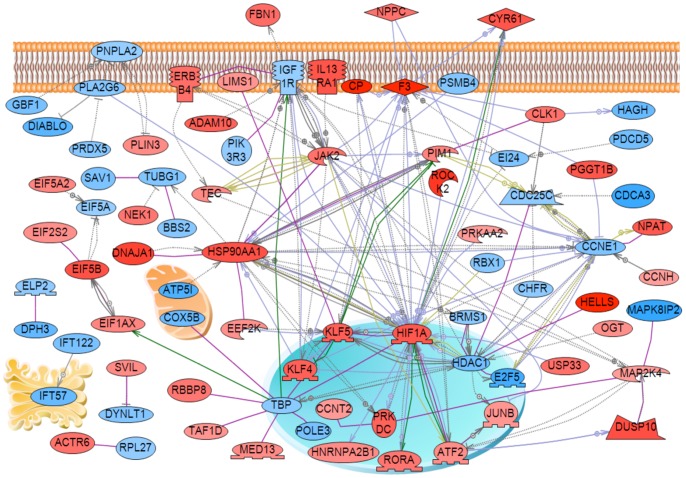
Gene network of Sertoli cell differentially expressed genes. Genes having direct connectivity are presented with cellular localizations indicated.

A gene network analysis was performed on the 417 differentially expressed genes with an unbiased literature evaluation protocol using the Pathway Studio software. The down or up regulated genes associated and cellular localization are shown, Figure 4. The most highly interconnected genes were the *Igf1r, Jak2, Hsp90aa1, Hif1a* and *Ccne1*. As identified in the gene functional categories, Figure 2, a number of cellular processes are influenced by the gene network identified.

**Figure 5:**
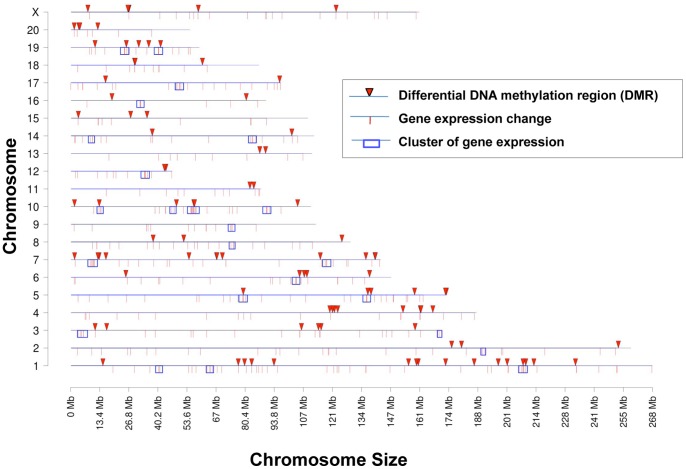
Chromosomal localization of differential DNA methylation regions (DMR), differential expressed genes, and clusters of gene expression. The chromosomal number and relative size are presented. The DMR (arrow), gene expression changes (line) and over-represented clusters of gene expression (box) are indicated.

The differentially regulated genes were mapped to the genome and shown in Figure 5. All chromosomes contained differentially expressed genes. The potential over-representation of gene clusters in specific chromosomal regions was examined as previously described [30]. Potential 2–5 megabase regions were examined for statistical over-representation of differentially expressed genes. These gene clusters are identified in Figure 5 and will be correlated to the epigenetic analysis described below.

### Sertoli Cell Transgenerational Epigenome

**Figure 6:**
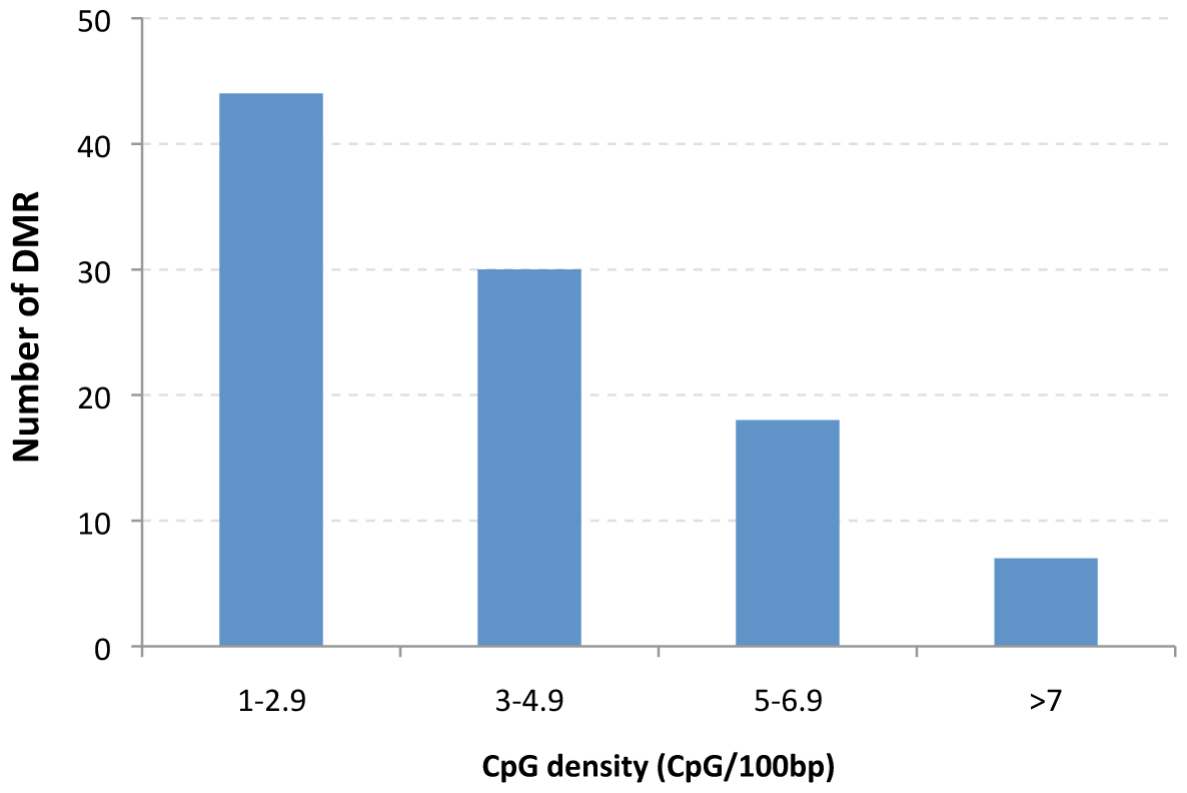
CpG density of the F3 generation vinclozolin lineage Sertoli cell DMR. The number of DMR and CpG density (CpG/100 bp) range are presented.

The F3 generation control and vinclozolin lineage Sertoli cell DNA was used in a methylated DNA immunoprecipitation (MeDIP) and genome wide promoter tiling array (Chip) analysis (MeDIP-Chip) [8] as described in the Methods. A comparative hybridization of the F3 generation control and vinclozolin lineage Sertoli cell MeDIP samples identified differential DNA methylation regions (DMR), as previously described [8]. The MeDIP-Chip analysis identified 101 DMR and the chromosomal locations of the DMR are shown in Figure 5 and Supplemental Table S2. All chromosomes contained DMR. Interestingly, none of the Sertoli cell DMR identified were in common with the previously identified sperm DMR [8]. In the analysis of sperm DMR previously reported [8], two genomic features were identified. The first was a consensus DNA sequence localization in the ∼600 bp region of the DMR termed “environmental induced DNA methylation motif 1” (EDM1) [8]. This DNA sequence motif is a >20 bp sequence motif associated with 68.8% of the DMR identified in sperm [3], [8], as described in the Methods. The EDM1 motif was found in 7.1% of the Sertoli cell DMR identified, which was not statistically different from a computer generated random promoter region set with a 16.1% incidence of occurrence. Therefore, the EDM1 motif genomic feature found in sperm DMR was not associated with the Sertoli cell DMR. The second genomic feature previously identified in sperm DMR was a low density CpG content of less than 10 CpG/100 bp [3], [8]. The number of CpG/100 bp is generally between 1–4 in the sperm DMR. The Sertoli cell DMR were also found to contain a low density of CpG (<10 CpG/100 bp), Figure 6. The majority of DMR had 1 or 2 CpG/100 bp. No DMR was found to have a CpG density greater than 10.6 CpG/100 bp. Therefore, the Sertoli cell DMR are similar to the Sperm DMR in that a CpG “desert” of low density CpG is a genomic feature involved [3], [8].

The MeDIP-Chip DMR data was confirmed with a select set of Sertoli cell DMR using an MeDIP-quantitative PCR (QPCR) analysis. The selected 26 DMR sites with 7 confirmations using the MeDIP-QPCR analysis are presented in Supplemental Figure S3. This analysis confirmed the MeDIP-Chip data for these DMR. A technical limitation to this analysis is that only <150 bp region within the ∼600–800 bp DMR can be examined, such that false negatives are common due to the inability to interrogate the entire DMR. Although the MeDIP-Chip data for a number of DMR were confirmed, a better technology is needed for future studies.

**Figure 7:**
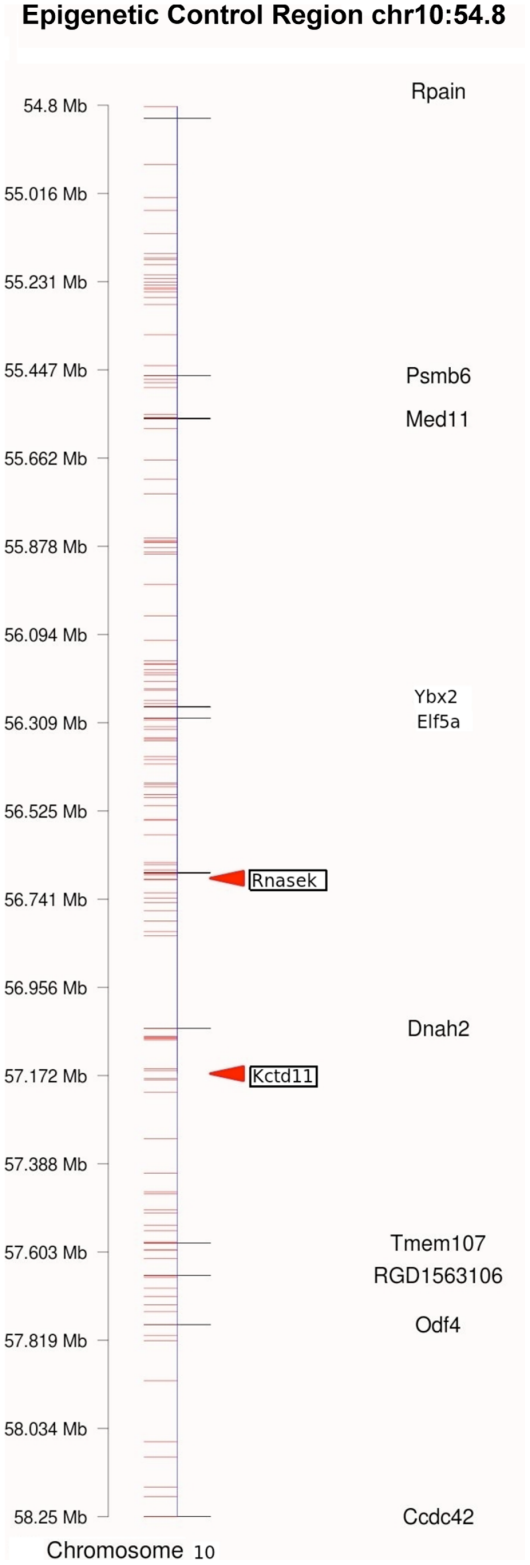
Example of an epigenetic control region (ECR) on chromosome 10. The chromosomal location in megabases is presented with all genes (horizontal lines) and regulated genes (gene symbols) listed. The DMR are listed in arrow heads.

A correlation of Sertoli cell differentially expressed genes with the DMR identified only two differentially expressed genes that also had a corresponding promoter DMR (Pdrx5 and Pole3), Supplemental Table S3. Therefore, the majority of differentially expressed genes did not contain a DMR for potential direct regulation of gene expression. Previously, we identified the potential presence of “epigenetic control regions” (ECR) that contain a statistically significant over-representation of differential expressed genes [30]. Gene clusters within 2–5 megabase regions that contain a statistically over-representation of differentially expressed genes are shown in Figure 5 and Supplemental Table S4. Six clusters had a DMR present within a 2 megabase window, Figure 5 and Supplemental Table S3. These regions may act as an ECR to regulate gene expression in the region as previously described [30]. An example of one potential ECR is presented in Figure 7. This ECR contained two DMR (*Rnase1a* and *Kctdll*) and had 10 differentially expressed genes within the approximately 5 megabase region shown. Therefore, 61 genes in the 16 potential DMR may be regulated as an ECR.

**Figure 8:**
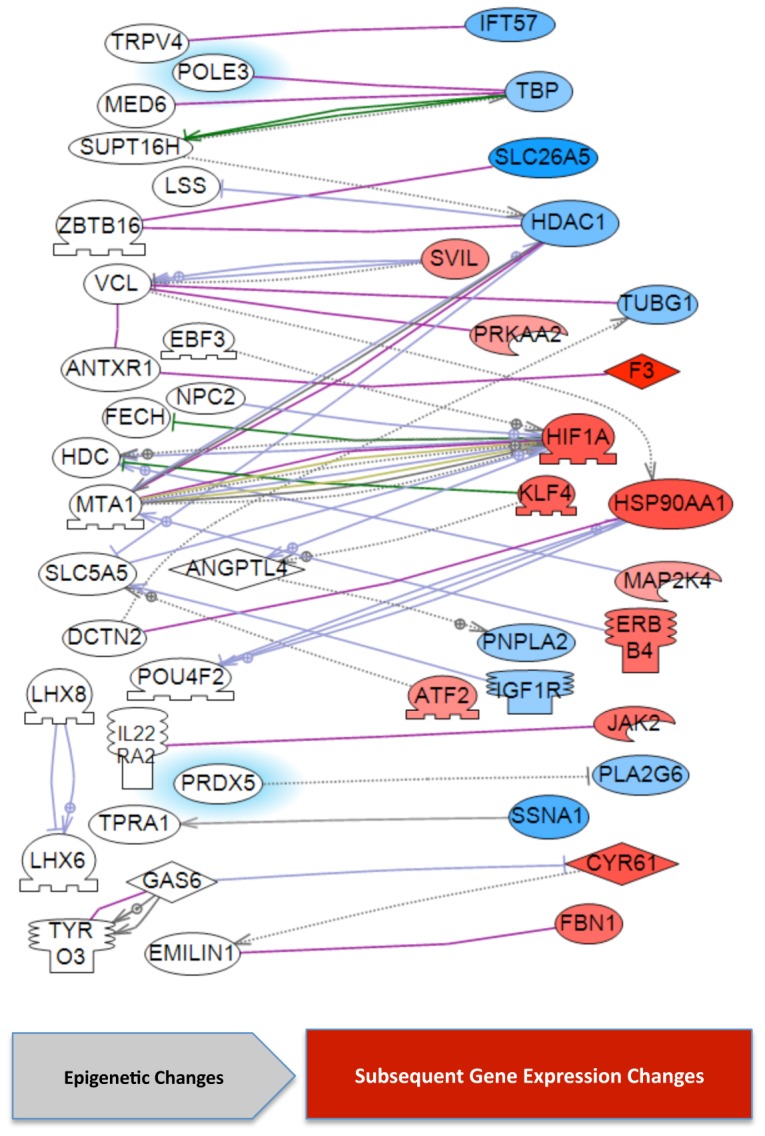
Gene interactions between DMR and differentially expressed genes in F3 generation vinclozolin lineage Sertoli cells. The white genes are DMR associated genes and the colored genes differentially expressed genes.

A correlation of potential distal gene expression identified 31 DMR that were within 2 megabase of 38 differentially regulated genes, Supplemental Table S3. Although the potential mechanism involved are unclear, distal regulation through mechanisms such as non-coding RNA have been demonstrated [30]. A final correlation used an unbiased literature based analysis to identify indirect gene interactions between the DMR and differentially expressed genes. The DMR associated genes that have been shown to interact with the Sertoli cell differentially expressed genes are shown in Figure 8. A potential correlation is shown between 25 DMR and 21 differentially expressed genes. Although a limited number of genes had direct regulation, a large number of the differentially expressed genes correlate to potential ECR, distal regulation sites or indirect gene interactions, Supplemental Table S3 and S4.

**Figure 9:**
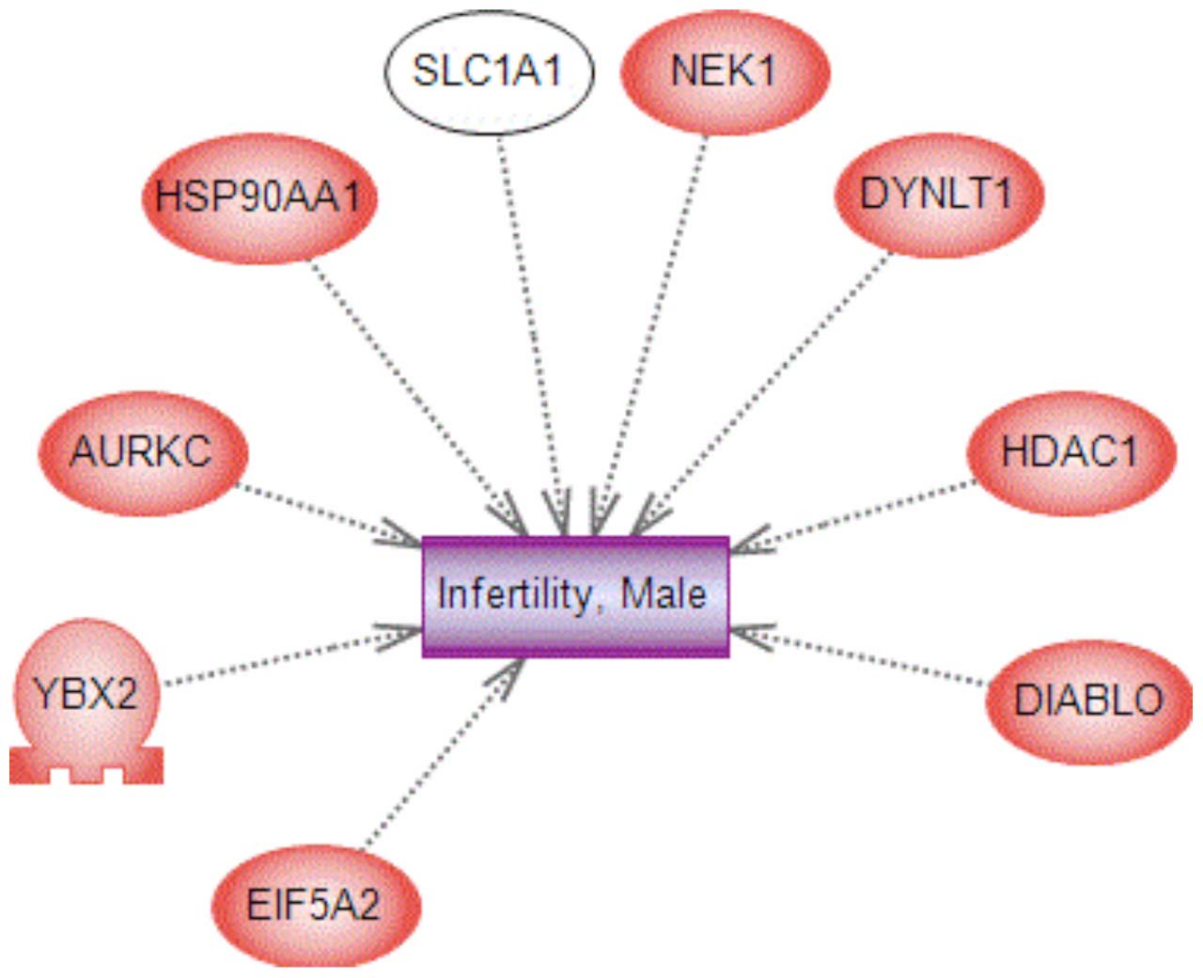
DMR and differentially expressed genes that correlate with male infertility/testis disease. The genes identified in the literature associated with male infertility and testis disease that correlate with F3 generation vinclozolin lineage DMR and differentially expressed genes are presented.

The 101 DMR associated genes and the 417 Sertoli cell differentially regulated genes were used to identify correlations with genes previously associated with male infertility. This analysis used a literature based procedure in the Pathway Studio software and results are shown in Figure 9. The correlation demonstrated one DMR and eight differentially expressed genes had a direct relationship with previously associated male infertility genes. Observations provide additional insights into the molecular etiology of male infertility.

## Discussion

The ability of environmental toxicants such as vinclozolin have previously been shown to promote the epigenetic transgenerational inheritance of adult onset disease such as male infertility [2], [12], [14]. Epigenetic transgenerational phenomena require the germ line transmission of a permanently modified epigenome (e.g. epimutation) [1], [26]. How this modified germline epigenome is translated into later life adult onset disease was investigated in the current study with a focus on testis biology and disease. Observations confirmed the ability of vinclozolin to promote F3 generation spermatogenic cell apoptosis, that has previously been correlated to adult onset male infertility [2], [12]. This transgenerational model system was used to evaluate the molecular etiology of male infertility. The Sertoli cell is the essential somatic cell known to support spermatogenesis [31], [32] and abnormal Sertoli cell function has been shown to correlate to testis disease [38], [39], [40]. Therefore, the Sertoli cell was the focus of the current study to assess how a germline epimutation may translate to male infertility.

The Sertoli cell transgenerational transcriptome induced in the F3 generation vinclozolin lineage identified 417 differentially expressed genes. All somatic cell types derived from the gamete having germline epimutations will have a transgenerational alteration in the transcriptome, as previously shown for 11 different male and female tissues [30] and granulosa cells [15]. Each tissue and somatic cell was found to have a unique transgenerational transcriptome. The Sertoli cell transcriptome identified in the current study supports the concept of transgenerational alteration of individual somatic cell types. Cell types or tissues sensitive to this alteration in the transcriptome will be susceptible to develop disease [1], [29]. The vinclozolin lineage Sertoli cell transgenerational transcriptome was found to have both up and down regulated genes with predominant cellular pathways and gene functional categories affected. A gene network analysis identified specific genes that influence a number of different signaling pathways and processes.

Considering the role of Sertoli cells in the maintenance of spermatogenic cell development, a critical pathway was identified that directly correlates with germ cell viability. Sertoli cells acquire glucose on their basal surface and convert this to pyruvate and lactate that is then secreted and provided to germ cells, sequestered within the blood-testis barrier, as a primary energy source [35], [36]. Previous studies have shown abnormal pyruvate or lactate production or transport promotes the degeneration of spermatogenic cells through the process of apoptosis [36], [47], [48], [49]. Therefore, the transgenerational alteration in pyruvate or lactate production by the Sertoli cells provides a direct mechanism for the induction of spermatogenic cell apoptosis observed in the F3 generation vinclozolin lineage males. Although other differentially expressed genes and cellular processes are likely involved, the abnormal pyruvate or lactate production directly correlates with the transgenerational adult onset male infertility observed [2], [12].

The F3 generation vinclozolin lineage Sertoli cells also had an altered transgenerational epigenome. The genome-wide promoter analysis identified 101 differential DNA methylation regions (DMR) that previously have been termed epimutations [1], [8], [29]. The Sertoli cell transgenerational DMR were localized on the genome and genomic features analyzed. Interestingly, the previously identified EDM1 DNA sequence motif shown to be associated with the sperm DMR [8], was not associated with the Sertoli cell DMR. Observations suggest different molecular mechanisms may be involved in the germline versus somatic cell DMR generation and function. In contrast, the low density of CpG (<10 CpG/100 bp) identified in the sperm DMR was also observed in the Sertoli cell DMR. As previously discussed [3], [8], these “CpG deserts” may have evolved as critical regulatory epigenetic sites. The high mutation rates observed in CpG sites [50] suggests evolutionarily over time that regions of the genome develop low density CpG (e.g. CpG deserts). In the event a CpG cluster is regulatory in these deserts then evolutionary pressure maintains the presence of the clusters in these desert regions. Observations suggest that this same genomic feature observed in sperm of a low density <10 CpG/100 bp in the 600–800 bp DMR also exists in somatic cells (Sertoli cells). Although this CpG desert genomic feature was similar, the specific DMR in sperm were found to have no overlap with the Sertoli cell DMR identified. Therefore, the DMR in somatic cells are likely unique to the cell type and generated from a specific cascade of epigenetic alterations derived from the germline and early embryo.

A correlation of the transgenerational Sertoli cell transcriptome with the epigenome identified potential distinct regulatory mechanisms. Interestingly, only two genes (Prdx5 and Pole3) had a direct correlation to suggest the potential for the DMR to directly regulate the promoter and expression of the adjacent gene. Therefore, the majority of the altered transcriptome and DMR act through different mechanisms. Distal regulation was considered and approximately 10% of the differentially expressed genes were found within 2 megabases of a DMR. Such distal regulation could involve mechanisms such as non-coding RNA [30], [51]. Perhaps the most interesting correlation is the role of epigenetic control regions (ECR) [30]. Previously we demonstrated that differentially expressed genes can be statistically over-represented and localized in gene clusters on the genome [30]. When an epigenetic regulatory site such as a DMR is also localized in the 2–5 megabase gene cluster then the region may be regulated through an ECR. The initial epigenetic regulatory regions identified were imprinting control regions (ICR) [51], [52]. A defined DNA methylation region was found to regulate a non-coding RNA that distally for several megabase regulated the expression of multiple genes [51]. The ICR are likely a subset of a large number of such ECR that allow for a limited number of epigenetic regulatory sites to influence the expression of a large number of genes [30]. The F3 generation Sertoli cell differentially expressed genes clustered into 16 different potential regions and a number of these directly correlated to the location of DMR identified. A large number of genes were regulated within these potential ECR. Therefore, a combination of direct regulation, distal regulation, and ECR are likely involved in the Sertoli cell transgenerational transcriptome identified. In addition, indirect regulatory links between DMR associated genes and the differentially expressed genes were identified. The epigenetic regulation of somatic cell gene expression and role of epigenetic transgenerational inheritance needs to be further elucidated.

The current study used a transient exposure during the developmental period of germ cell epigenetic programming and gonadal sex determination [1], [2], [3]. The disease observed in the F1 generation offspring are due to direct exposure of the fetal somatic cells and not to a germline mediated transgenerational mechanism observed in the F3 generation. Although a comparison of the F1 and F3 generation control versus vinclozolin lineage Sertoli cells would be interesting, there is no anticipated correlations due to the distinct molecular mechanism involved. Similarly, direct exposure of an adult male can alter the epigenetic programming in spermatogenic cells to subsequently effect the disease in the F1 generation offspring [1], but these direct exposures have not previously been shown to promote transgenerational effects to subsequent generations. Therefore, the current study focused on the F3 generation to investigate the epigenetic transgenerational inheritance of disease mechanisms. Future studies to investigate the differences with the direct exposure fetal or adult male effects on the F1 generation will be interesting, but have distinct mechanism with the observations presented in the current study.

Male infertility in the human population has now increased to affect over 10% of the population [43]. A number of studies suggest environmental factors such as toxicants likely have a significant role in the etiology of male infertility [53], [54]. Direct exposure toxicity of some of these agents for testis function has been observed [2], [3], but how such exposures may promote later life disease has not been clarified. The current study used a rat model involving an environmental toxicant (vinclozolin) induced epigenetic transgenerational inheritance of testis disease, that appears in the vast majority of F3 generation males, to study the molecular etiology of male infertility. Observations demonstrate a transgenerational effect on the Sertoli cell transcriptome and epigenome that impacts critical cellular processes involved in testis function. The pyruvate/lactate pathway is critical for spermatogenesis and the transgenerational Sertoli cell transcriptome suggests that abnormal pyruvate/lactate production could directly promote the spermatogenic cell apoptosis observed. Further studies are needed to directly test this mechanism, but previous literature supports the critical role of pyruvate/lactate in spermatogenic cell viability. A correlation of the transgenerational Sertoli cell DMR and differentially expressed genes with previously identified genes associated with male infertility [55], [56] identified nine correlated genes. Therefore, in addition to the abnormal pyruvate/lactate metabolism the abnormal expression of these genes is known to be associated with male infertility.

The molecular etiology of male fertility identified suggests environmental toxicant exposure of a gestating female at the critical period of gonadal sex determination promotes an abnormal programming of the germ line epigenome (DNA methylation) that is transmitted transgenerationally to subsequent generations and promotes adult onset testis disease. This is correlated to abnormal Sertoli cell function and reduction in pyruvate/lactate production, as well as other critical gene expression abnormalities, to promote spermatogenic cell apoptosis and male infertility. The degree environmental induced epigenetic transgenerational inheritance of testis disease is associated with human male infertility now needs to be assessed. The rat model used clearly demonstrates that these exposures and molecular events are associated with the high incidence of male infertility transgenerationally. Combined observations clearly establish the role of transgenerational alterations in a somatic cells transcriptome and epigenome as a likely component of the etiology of environmentally induced epigenetic transgenerational inheritance of adult onset disease.

## Materials and Methods

### Animals and Treatments

Hsd:Sprague Dawley®™SD®™ female and male rats of an outbred strain (Harlan) were maintained in ventilated (up to 50 air exchanges/hour) isolator cages (cages with dimensions of 10 ¾” W×19 ¼ “D×10 ¾” H, 143 square inch floor space, fitted in Micro-vent 36-cage rat racks; Allentown Inc., Allentown, NJ) containing Aspen Sani chips (pinewood shavings from Harlan) as bedding, on a 14 h light: 10 h dark regimen, at a temperature of 70 F and humidity of 25% to 35%. Rats were fed ad libitum with standard rat diet (8640 Teklad 22/5 Rodent Diet; Harlan) and *ad libitum* tap water for drinking. At pro-estrus as determined by daily vaginal smears, the female rats (90 days of age) were pair-mated with male rats (120 days). On the next day, the pairs were separated and vaginal smears were examined microscopically. If sperm were detected (day 0) the rats were tentatively considered pregnant. Vaginal smears were continued for monitoring diestrus status in these rats until day 7. Pregnant rats for the treatment group (six different gestating females for each group) were given daily intraperitoneal injections of vinclozolin (100 mg/kg BW/d; Chem Service, West Chester, PA) and an equal volume of sesame oil (Sigma) on days E8 through E14 of gestation; Vinclozolin was dissolved in DMSO (Sigma). Pregnant rats for the control group were given daily intraperitoneal injections of DMSO (100 ul/kg BW/d) and an equal volume of sesame oil (Sigma) on days E8 through E14 of gestation [45]. The pregnant female rats treated with vinclozolin were designated as the F0 generation. All experimental protocols for the procedures with rats were pre-approved by the Washington State University Animal Care and Use Committee (IACUC approval # 02568-029).

### Breeding for F1, F2, and F3 Generations

The offspring of the F0 generation were the F1 generation. The F1 generation offspring were bred to other F1 animals of the same treatment group to generate an F2 generation and then F2 generation animals bred similarly to generate the F3 generation animals. No sibling or cousin breeds were performed so as to avoid inbreeding. Note that only the original F0 generation pregnant females were injected with vinclozolin or vehicle. The animals within a group were bred to optimize the transgenerational phenotype.

### Measurement of Testicular Apoptotic Cells by TUNEL Analysis

Testis sections were examined by terminal deoxynucleotidyl transferase-mediated dUTP nick end labeling (TUNEL) assay (*in situ* cell death detection kit, Fluorescein, Roche Diagnostics, Mannheim, Germany) as per the manufacturer’s protocols. Sections were deparaffinized and rehydrated through alcohol series. They were deproteinized by Proteinase K (20 mg/ml; Invitrogen, Carlsbad, CA), washed with PBS and then 25 µl of the enzyme-label solution mix was applied and incubated at 37°C for 90 min. After PBS washes, slides were mounted and kept at 4°C until examination with a fluorescent microscope using dark field. Both testis sections of each slide were microscopically examined to identify and to count apoptotic germ cells by the bright fluorescence.

### Sertoli Cell Preparation

Sertoli cells were isolated from the testes of 20-day-old rats (P20) using a sequential enzymatic digestion procedure previously described [57]. This pubertal period allows the optimum purity cells prior to disease onset. Three pools of P20 Sertoli cells were produced per treatment, with each pool containing cells from 2 to 6 animals. In brief, decapsulated testes were minced with razor blades and then tissue fragments were digested with trypsin (1.5 mg/ml, Life Technologies, Gaithersburg, MD) to remove the interstitial cells. This was followed by incubation with collagenase (1 mg/ml type I, Sigma) for removal of peritubular cells and then hyaluronidase (1 mg/ml, Sigma) for removal of germ cells. The purity of the Sertoli cell preparations were determined by immunohistochemistry to be >98% [57]. Final Sertoli cell pellets were then resuspended in 1 ml Trizol™ (Invitrogen) for further RNA and DNA extractions.

### RNA Extraction and Microarray Transcriptome Analysis

Messenger RNA was isolated using the Trizol™ (Invitrogen) method as per the manufacturer protocol. Messenger RNA was independently extracted from 3 pools of Sertoli cells (i.e. 3 biological replicas) per treatment. The mRNA processing and hybridizations were performed at the Genomics Core Laboratory, Center for Reproductive Biology, Washington State University, Pullman, WA using standard Affymetrix reagents and protocol. Briefly, mRNA was transcribed into cDNA with random primers, then cRNA was transcribed from the cDNA, and from that, single-stranded sense DNA was synthesized which was fragmented and labeled with biotin. Biotin-labeled fragmented ssDNA was then hybridized to the Rat Gene 1.0 ST microarrays containing more than 27,000 transcripts (Affymetrix, Santa Clara, CA, USA). Hybridized chips were scanned on an Affymetrix Scanner 3000. CEL files containing raw data were then pre-processed and analyzed with Partek Genomic Suite 6.5 beta software (Partek Incorporated, St. Louis, MO) using an RMA and GC-content adjusted algorithm (Supplemental Figure S1). The signals from an average of 28 different probes for each transcript were compared to give a single value. Two-way ANOVA was performed between the Sertoli cell transcriptomes from vinclozolin-lineage and controls. One factor of variation was treatment and the other was batch effect. Corrections were made for Sertoli cell preparation date batch effect by the Partek software according to the Methods of Moments [58]. The selection of the gene expression change was based on the expression change between vinclozolin and control lineage Sertoli cells limited to p-values <0.05, expression fold change >1.2, and the mean difference between vinclozolin and control un-logged signals >10. A higher stringency cut off (>1.5 fold change) was not utilized since many biological effects are observed with 20% alterations in gene expression so a more genome wide view of the transcriptome is identified with the stringency utilized. CEL files from this study have been deposited with the NCBI gene expression and hybridization array data repository (GEO, http://www.ncbi.nlm.nih.gov/geo, GEO # pending) and can be also accessed through www.skinner.wsu.edu. For gene annotation, the Affymetrix annotation file RaGene1_0stv1.na31.rn4.transcript.csv was used unless otherwise specified.

### Pathway and Gene Network Analysis

Known functional relationships among the F3 generation differentially expressed genes were identified using the KEGG pathways from the University of Kyoto (Japan) Encyclopedia for Genes and Genome website (http://www.genome.jp/kegg/) and Pathway Express (http://vortex.cs.wayne.edu) [59]. Functional relationships among the F3 generation differentially expressed genes and genes with changes in DNA methylation were interrogated using Pathway Studio software (Ariadne, Genomics Inc. Rockville MD), using an unbiased, automated survey of published scientific literature (Global Literature Analysis). This analysis identifies functional relations among genes, such as direct binding, up-regulation or down-regulation and also builds sub-networks of genes and cellular processes based on their inter-connections.

### DNA Extraction and Methylated DNA Immunoprecipitation (MeDIP)

DNA was isolated using the Trizol™ (Invitrogen) method as per the manufacturer protocol, from the same Sertoli cell Trizol™ preparations that were used for RNA isolations. Therefore, three independent DNA Trizol™ fractions from Sertoli cells per group were used to obtain three different biological replicates of DNA samples from each of the two treatment groups. Each of these DNA samples were then used for methylated DNA immunoprecipitation (MeDIP). MeDIP was performed as follows: 6 mg of genomic DNA was subjected to a series of three 20 pulse sonications at 20% amplitude. The appropriate fragment size (200–1000 ng) was verified through 2% agarose gels. The sonicated genomic DNA was resuspended in 350 ul TE and denaturated for 10 min at 95°C and then immediately placed on ice for 5 min; 100 ul of 5X IP buffer (50 mM Na-phosphate pH7, 700 mM NaCl, 0.25% Triton X-100) was added to the sonicated and denatured DNA. An overnight incubation of the DNA was performed with 5 ug of antibody anti-5-methylCytidine monoclonal from Diagenode S.A (Denville, NJ) at 4°C on a rotating platform. Protein A/G beads from Santa Cruz (Santa Cruz, CA) were prewashed on PBS-BSA 0.1% and resuspended in 40 ul 1X IP buffer. Beads were then added to the DNA-antibody complex and incubated 2 h at 4°C on a rotating platform. Beads bound to DNA-antibody complex were washed 3 times with 1 ml 1X IP buffer; washes included incubation for 5 min at 4°C on a rotating platform and then centrifugation at 6000 rpm for 2 min. Beads-DNA-antibody complex were then resuspended in 250 ul digestion buffer (50 mM Tris HCl pH 8, 10 mM EDTA, 0.5% SDS) and 3.5 ul of proteinase K (20 mg/ml) was added to each sample and then incubated overnight at 55°C on a rotating platform. DNA purification was performed first with phenol and then with chloroform:isoamyl alcohol. Two washes were then performed with 70% ethanol, 1 M NaCl and glycogen. MeDIP selected DNA was then resuspended in 30 ul TE buffer. Whole-genome amplification was then performed with the WGA2 kit (Sigma-Aldrich) on each MeDIP sample to be used in the microarray comparative hybridization analysis.

### Tilling Array and MeDIP-Chip Bioinformatic and Statistical Analyses

Roche Nimblegen’s Rat DNA Methylation 3×720 K CpG Island Plus RefSeq Promoter Array was used, which contains three identical sub-arrays, with 713,670 probes per sub-array, scanning a total of 15,287 promoters (3,880 bp upstream and 970 bp downstream from transcription start site). Probe sizes range from 50–75 mer in length with the median probe spacing of 100 bp. Three different comparative (amplified MeDIP vs. amplified MeDIP) hybridizations experiments included in three sub-arrays were performed by Nimblegen. Each comparative hybridization experiment contained one biological replicate of Sertoli cell Whole Genome Amplified-MeDIP-DNA sample from each lineage-treatment. Samples from experimental groups were labeled with Cy3 and MeDIP DNA samples from the control groups were labeled with Cy5. For each comparative hybridization experiment, raw data from both the Cy3 and Cy5 channels were imported into R (R Development Core Team (2010), R: A language for statistical computing, R Foundation for Statistical Computing, Vienna, Austria. ISBN 3-900051-07-0, URL http://www.R-project.org), checked for quality and converted to MA values (M = Cy5-Cy3; A = (Cy5+Cy3)/2). The following normalization procedure was conducted. Within each array, probes were separated into groups by GC content and each group was separately normalized, between Cy3 and Cy5 using the loess normalization procedure. This allowed for GC groups to receive a normalization curve specific to that group. After each array had its CG groups normalized within the array, the arrays were then normalized across arrays using the A quantile normalization procedure. Following normalization each probe within each array was normalized and M values were replaced with the median value of all probe normalized M values across all arrays within a 600 bp window. If the number of probes present in the window was less than 3, no value was assigned to that probe. Each probe’s A values were likewise normalized using the same procedure. Following normalization each probe’s M value represents the median intensity difference between vinclozolin generation and control generation of a 600 bp window. Significance (p<10^−5^) was assigned to probe differences between treatment-generation samples and control generation samples by calculating the median value of the intensity differences as compared to a normal distribution scaled to the experimental mean and standard deviation of the normalized M. A Z-score and P-value were computed for each probe from that distribution. The statistical analysis was performed in pairs of comparative IP hybridizations between treatment-lineage (T) and control-lineage (C). T1-C1 and T2-C2 gave 715 sites; T1-C1 and T3-C3 gave 633 sites; T2-C2 and T3-C3 gave 807 sites. In order to assure the reproducibility of the candidates obtained, only the candidates showing significant changes in all three of the paired comparisons were chosen as having a significant change in DNA methylation between the experimental group and controls. This is a very stringent approach to select for changes, since it only considers those changes repeated in all paired analyses.

Clustered Regions of interest were then determined by combining consecutive probes within 600 bases of each other, and based on whether their mean M values were positive or negative, with significance p-values less than 10^−5^. The statistically significant differential DNA methylated regions were identified and p-value associated with each region presented. Each region of interest was then annotated for gene and CpG content. This list was further reduced to those regions with an average intensity value exceeding 9.5 (log scale) and a CpG density ≥1 CpG/100 bp. The web-based tool FIMO (Found Individual Motifs Occurrences) was used for determining the incidence of motifs in sets of sequences [60].

### Chromosomal Location of Gene Expression Clusters

An R-code was developed to find chromosomal locations of ECRs (Figure 8). A 2 megabase sliding window with 50,000 bases interval was used to find the associated genes in each window. Then a Z-test statistical analysis with p<0.05 was used on these windows to find the ones with over-representation of differentially expressed genes. The consecutive windows with over-represented genes were merged together to form clusters of genes which we named ECR regions. Typical ECR regions ranged from 2–5 megabase.

## Supporting Information

Figure S1 **Microarray histograms for each array and box plot for The F3 generation control and vinclozolin lineage Sertoli cell samples.** Array data was pre-processed with RMA and GC-content adjusted algorithm in Partek GS program. The y-axis presents the new hybridization signal and box plots the mean ± SEM.
(PDF)

Figure S2 **Cellular signaling and process pathways impacted by differentially expressed genes from KEGG (see Methods).** a) Proteosome, b) Nucleotide excision repair, c) MTOR signaling pathway, d) RNA transport.
(PDF)

Figure S3 **Quantitative PCR of F3 generation Sertoli cell MeDIP for selected genes.** The fold change (2∧-deltaC+) between the control and vinclozolin lineage Sertoli cell MeDIP samples is presented with the black bars indicating samples with a significant difference (p<0.05).
(PDF)

Table S1 **Differentially expressed genes from F3 generation vinclozolin lineage Sertoli cells as compared to control lineage cells (416 genes & ESTs).**
(PDF)

Table S2 **Differential DNA methylation regions (DMR) in F3 generation vinclozolin lineage Sertoli cells.**
(PDF)

Table S3 **Relations between vinclozolin-induced transgenerational DMR and expression changes in Sertoli cells.**
(PDF)

Table S4 **Clusters of transgenerational changes in gene expression and their relation to DMR in vinclozolin-lineage F3 Sertoli cells.**
(PDF)
