# Supplementary figures and images for: Environmentally Induced Epigenetic Transgenerational Inheritance of Altered Sertoli Cell Transcriptome and Epigenome: Molecular Etiology of Male Infertility
Source: PLoS One. 2013 Mar 28;8(3):e59922. doi: 10.1371/journal.pone.0059922 (PMC3610698; doi:10.1371/journal.pone.0059922)

Supplemental Figure S1 (Color)

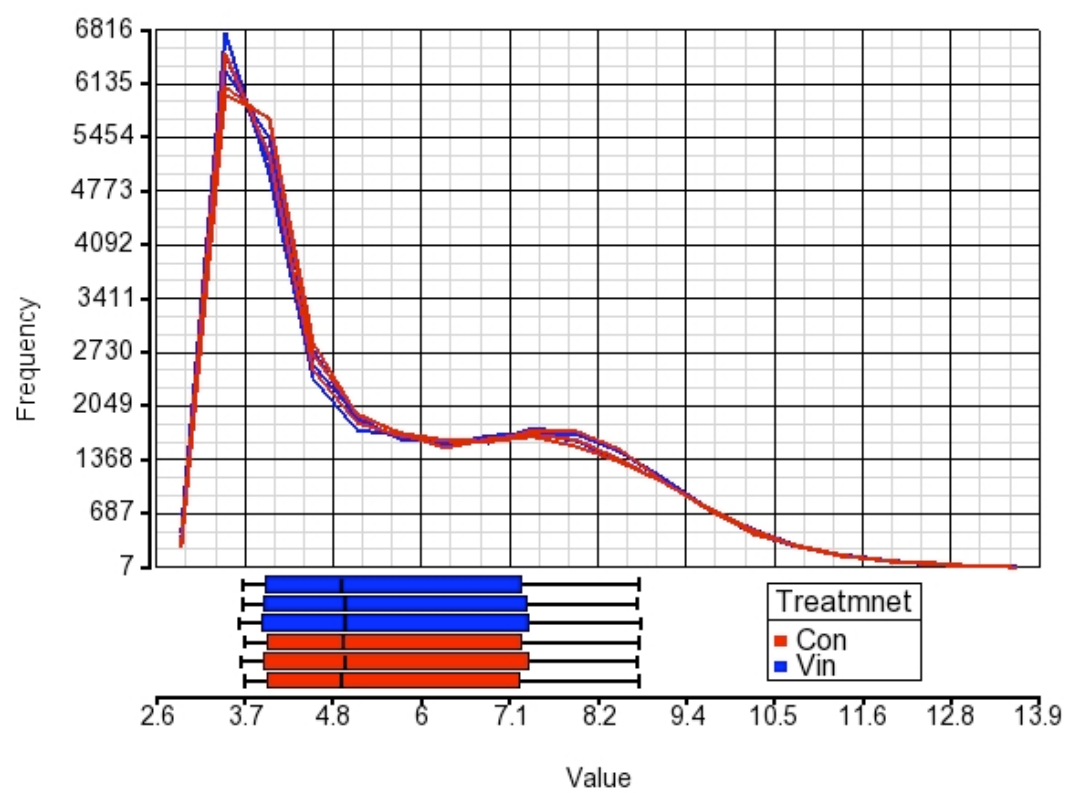

Supplement: Figure S1 — Microarray histograms for each array and box plot for The F3 generation control and vinclozolin lineage Sertoli cell samples. Array data was pre-processed with RMA and GC-content adjusted algorithm in Partek GS program. The y-axis presents the new hybridization signal and box plots the mean ± SEM. (PDF) [file pone.0059922.s001.pdf]

**Supplemental Figure S3**

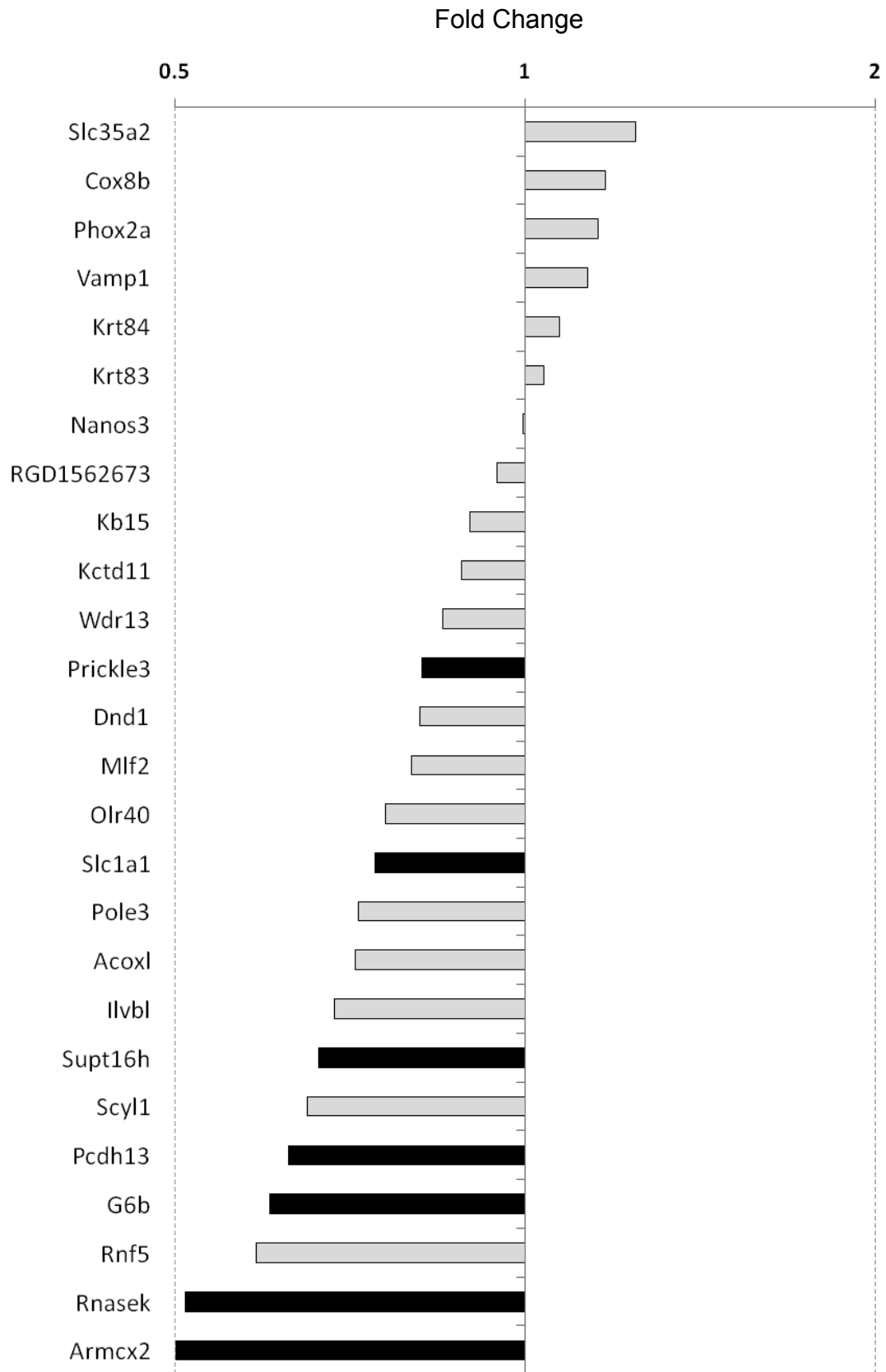

Supplement: Figure S3 — Quantitative PCR of F3 generation Sertoli cell MeDIP for selected genes. The fold change (2∧-deltaC+) between the control and vinclozolin lineage Sertoli cell MeDIP samples is presented with the black bars indicating samples with a significant difference (p<0.05). (PDF) [file pone.0059922.s003.pdf]
