# Supplementary material for: Environmentally Induced Epigenetic Transgenerational Inheritance of Altered Sertoli Cell Transcriptome and Epigenome: Molecular Etiology of Male Infertility
Source: PLoS One. 2013 Mar 28;8(3):e59922. doi: 10.1371/journal.pone.0059922 (PMC3610698; doi:10.1371/journal.pone.0059922)

Supplemental Figure S2 (Color)

a)

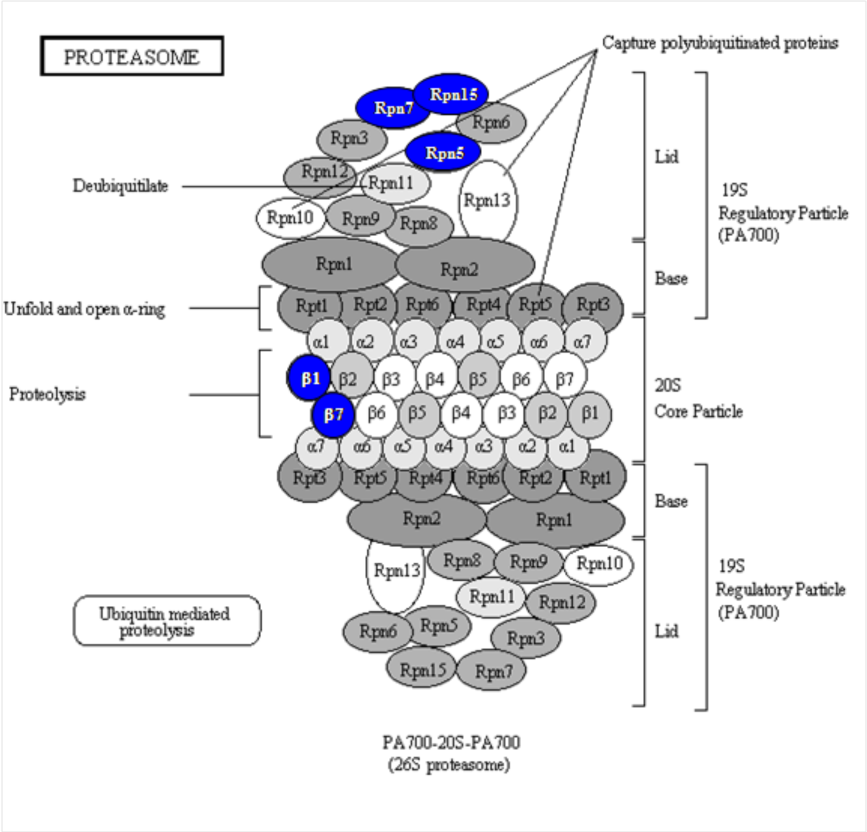

b)

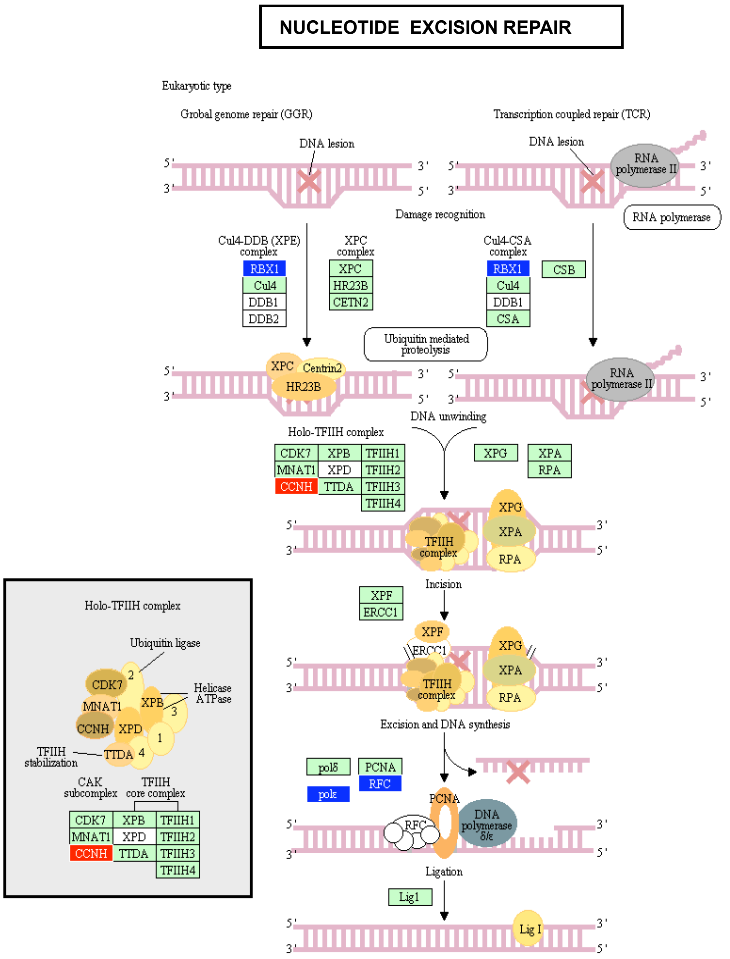

Supplemental Figure S2 (Cont.)

c)

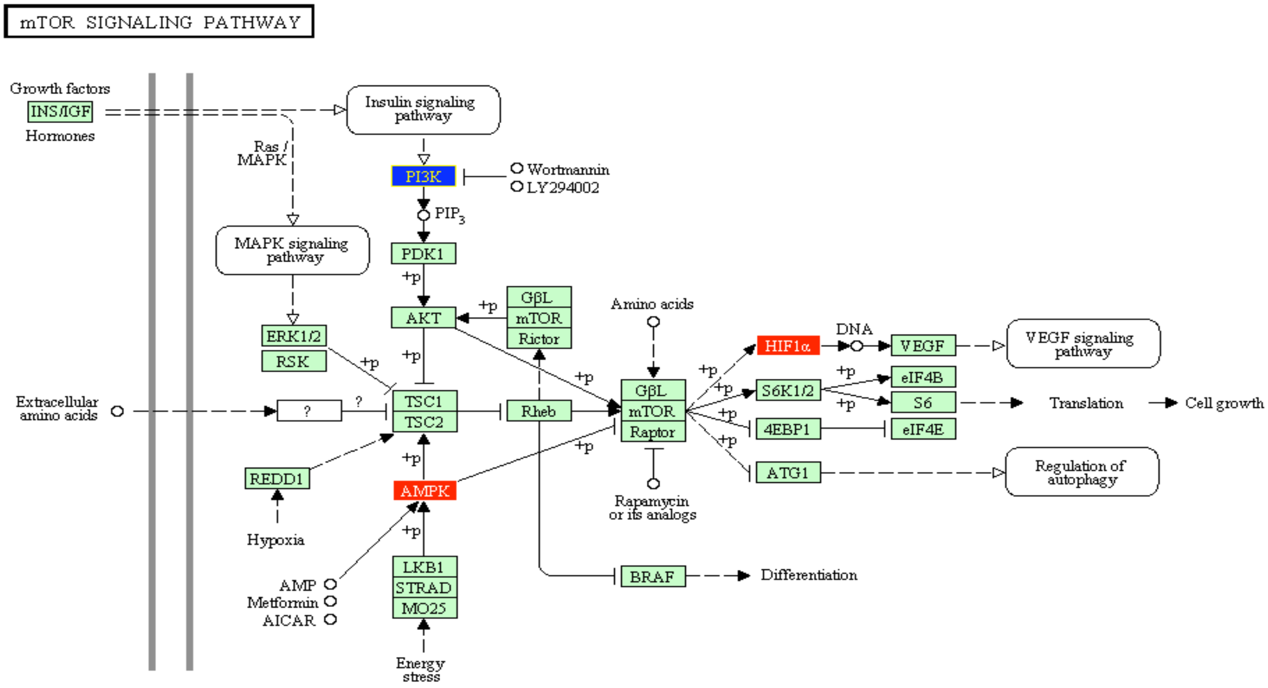

d)

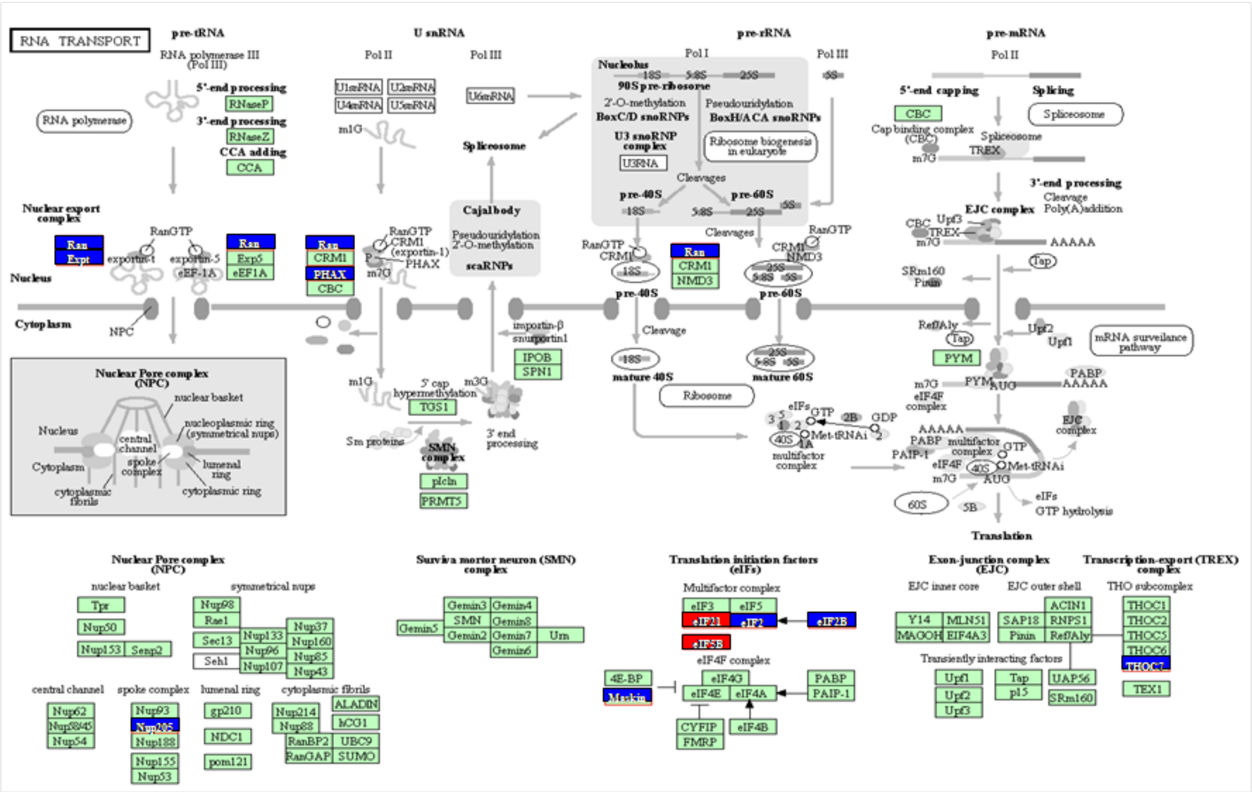

Supplement: Figure S2 — Cellular signaling and process pathways impacted by differentially expressed genes from KEGG (see Methods). a) Proteosome, b) Nucleotide excision repair, c) MTOR signaling pathway, d) RNA transport. (PDF) [file pone.0059922.s002.pdf]
