# Supplementary material for: Environmentally Induced Epigenetic Transgenerational Inheritance of Altered Sertoli Cell Transcriptome and Epigenome: Molecular Etiology of Male Infertility
Source: PLoS One. 2013 Mar 28;8(3):e59922. doi: 10.1371/journal.pone.0059922 (PMC3610698; doi:10.1371/journal.pone.0059922)
Supplement: Table S1 — Differentially expressed genes from F3 generation vinclozolin lineage Sertoli cells as compared to control lineage cells (416 genes & ESTs). (PDF) [file pone.0059922.s004.pdf]

**Supplemental Table S1.**
**Differentially expressed genes from F3 generation vinclozolin lineage Sertoli cells as compared to control lineage cells (416 genes & ESTs)**

| Gene Symbol | GenBank, Reference Sequence | Ratio (Vin/Con) | mean_dif (Vin-Con) | ProbeSet ID | Gene Title |
|---|---|---|---|---|---|
| **Apoptosis** | | | | | |
| Card6 | NM_001106413 | **1.27** | **16** | 10821685 | caspase recruitment domain family, member 6 |
| Diablo | NM_001008292 | **0.69** | **-271** | 10758432 | diablo homolog (Drosophila) |
| Lins1 | XM_218748 | **0.80** | **-19** | 10707837 | lines homolog 1 (Drosophila) |
| Pdcd2l | NM_001109544 | **0.78** | **-22** | 10720982 | programmed cell death 2-like |
| Pdcd5 | NM_001106247 | **0.74** | **-76** | 10721120 | programmed cell death 5 |
| **Cell Cycle** | | | | | |
| Ccne1 | NM_001100821 | **0.77** | **-14** | 10721176 | cyclin E1 |
| Ccnh | NM_052981 | **1.20** | **41** | 10812363 | cyclin H |
| Ccnt2 | NM_001107171 | **1.21** | **157** | 10767280 | cyclin T2 |
| Ccny | XM_341545 | **0.81** | **-60** | 10798952 | cyclin Y |
| Cdc25c | NM_001107396 | **0.81** | **-21** | 10803824 | cell division cycle 25 homolog C (S. pombe) |
| Cdca3 | NM_001007648 | **0.63** | **-16** | 10858707 | cell division cycle associated 3 |
| Cenpn | NM_001008366 | **0.70** | **-46** | 10808197 | centromere protein N |
| Cspp1 | XM_342793 | **1.33** | **61** | 10875064 | centrosome and spindle pole associated protein 1 |
| Chfr | NM_001009258 | **0.81** | **-31** | 10763116 | checkpoint with forkhead and ring finger domains |
| Pot1b | XM_001073777 | **1.36** | **14** | 10931288 | protection of telomeres 1B |
| Rbbp8 | NM_001134417 | **1.36** | **56** | 10800173 | retinoblastoma binding protein 8 |
| Syce2 | XM_344745 | **0.82** | **-41** | 10810040 | synaptonemal complex central element protein 2 |
| Tmem30c | NM_001105889 | **0.78** | **-15** | 10750701 | transmembrane protein 30C |
| Tacc3 | NM_001004424 | **0.78** | **-19** | 10777770 | transforming, acidic coiled-coil containing protein 3 |
| **Cytoskeleton-ECM** | | | | | |
| Actr10 | NM_001009602 | **0.74** | **-251** | 10885031 | actin-related protein 10 homolog (S. cerevisiae) |
| Actr6 | NM_001108081 | **1.39** | **211** | 10901771 | ARP6 actin-related protein 6 homolog (yeast) |
| Dnah2 | XP_220603 | **0.71** | **-14** | 10743989 | dynein, axonemal, heavy chain 2 |
| Eml5 | AY445136 | **1.50** | **78** | 10891561 | echinoderm microtubule associated protein like 5 |
| Esco1 | NM_001126299 | **1.67** | **121** | 10767013 | establishment of cohesion 1 homolog 1 (S. cerevisiae) |
| Kif27 | NM_198050 | **0.73** | **-27** | 10793846 | kinesin family member 27 |
| Lysmd3 | NM_001009698 | **1.26** | **15** | 10812339 | LysM, putative peptidoglycan-binding, domain containing 3 |
| Odf4 | NM_001007670 | **0.65** | **-49** | 10743797 | outer dense fiber of sperm tails 4 |
| Rpain | NM_001033060 | **0.81** | **-16** | 10735400 | RPA interacting protein |
| Sgcb | XM_001056227 | **1.26** | **88** | 10772318 | sarcoglycan, beta (dystrophin-associated glycoprotein) |
| Svil | NM_001108416 | **1.27** | **14** | 10798794 | supervillin |
| Ttll13 | NM_001134962 | **0.67** | **-28** | 10708172 | tubulin tyrosine ligase-like family, member 13 |
| Tubg1 | NM_145778 | **0.78** | **-15** | 10738296 | tubulin, gamma 1 |
| RGD1560248 | XM_575134 | **1.30** | **40** | 10836212 | similar to formin-like 2 isoform B |
| **Development** | | | | | |
| Angel2 | NM_001135119 | **0.80** | **-63** | 10766709 | angel homolog 2 (Drosophila) |
| B9d1 | NM_001105786 | **0.77** | **-26** | 10734250 | B9 protein domain 1 |
| Bbs2 | NM_053618 | **0.79** | **-100** | 10806016 | Bardet-Biedl syndrome 2 |
| Bbs9 | XM_235942 | **0.72** | **-69** | 10908617 | Bardet-Biedl syndrome 9 |
| Bspry | NM_022261 | **0.66** | **-33** | 10869331 | B-box and SPRY domain containing |
| Cby1 | NM_145676 | **0.74** | **-43** | 10897698 | chibby homolog 1 (Drosophila) |
| Crbn | NM_001015003 | **1.57** | **172** | 10864525 | cereblon |
| Dynlt1 | NM_031318 | **0.79** | **-216** | 10717882 | dynein light chain Tctex-type 1 |
| Emg1 | NM_001107888 | **0.73** | **-115** | 10865452 | EMG1 nucleolar protein homolog (S. cerevisiae) |
| Fbn1 | NM_031825 | **1.41** | **44** | 10849327 | fibrillin 1 |
| Gtsf1 | NM_001079707 | **0.56** | **-61** | 10907722 | gametocyte specific factor 1 |
| Lrrc48 | NM_001013857 | **0.73** | **-83** | 10734045 | leucine rich repeat containing 48 |
| Lrrc56 | NM_001024902 | **0.69** | **-24** | 10712224 | leucine rich repeat containing 56 |
| Lrrc8b | NM_001107204 | **1.24** | **30** | 10775278 | leucine rich repeat containing 8 family, member B |
| Mospd1 | NM_001014107 | **1.45** | **20** | 10778399 | motile sperm domain containing 1 |
| Odz4 | XM_218927 | **1.42** | **23** | 10708695 | odz, odd Oz/ten-m homolog 4 (Drosophila) |

| | | | | | |
|---|---|---|---|---|---|
| Sav1 | NM_001097581 | **0.77** | **-28** | 10890397 | salvador homolog 1 (Drosophila) |
| Shfm1 | NM_001126090 | **0.70** | **-141** | 10860939 | split hand/foot malformation (ectrodactyly) type 1 |
| Ssna1 | NM_001107820 | **0.66** | **-110** | 10843396 | Sjogren syndrome nuclear autoantigen 1 |
| Surf4 | NM_001033868 | **0.81** | **-76** | 10843893 | surfeit 4 |
| Urb2 | NM_001135708 | **0.64** | **-174** | 10808764 | URB2 ribosome biogenesis 2 homolog (S. cerevisiae) |
| Vwa5a | NM_198755 | **1.52** | **51** | 10909210 | von Willebrand factor A domain containing 5A |
| Wbscr17 | NM_001025112 | **0.64** | **-24** | 10757940 | Williams-Beuren syndrome chromosome region 17 homolog (human) |
| LOC687994 | XM_001080810 | **1.27** | **48** | 10867109 | similar to 82-kD FMRP Interacting Protein |
| RGD1562629 | XM_001059612 | **1.22** | **51** | 10823252 | similar to neurobeachin |
| **DNA Repair** | | | | | |
| Mdc1 | NM_001166275 | **0.70** | **-85** | 10831041 | mediator of DNA damage checkpoint 1 |
| Rad54l | NM_001134960 | **0.75** | **-31** | 10871151 | RAD54 like (S. cerevisiae) |
| Rad9b | NM_001030042 | **0.79** | **-44** | 10758587 | RAD9 homolog B (S. cerevisiae) |
| Rad54b | XM_232785 | **0.73** | **-12** | 10867642 | RAD54 homolog B (S. cerevisiae) |
| **Electron Transport** | | | | | |
| Cox5b | NM_053586 | **0.73** | **-80** | 10922572 | cytochrome c oxidase subunit Vb |
| LOC305698 | XM_223806 | **1.31** | **13** | 10786123 | similar to Glutaredoxin-1 (Thioltransferase-1) (TTase-1) |
| LOC685322 | NM_001170465 | **0.72** | **-37** | 10778108 | similar to ubiquinol-cytochrome c reductase complex 7.2kDa protein isoform b |
| LOC688869 | NM_001145273 | **0.81** | **-133** | 10720721 | similar to cytochrome c oxidase, subunit VIb polypeptide 1 |
| Prdx5 | NM_053610 | **0.79** | **-48** | 10728240 | peroxiredoxin 5 |
| Asf1b | NM_001107160 | **0.64** | **-79** | 10810230 | ASF1 anti-silencing function 1 homolog B (S. cerevisiae) |
| Chd2 | NM_001107523 | **1.29** | **66** | 10722770 | chromodomain helicase DNA binding protein 2 |
| Gadd45g | NM_001077640 | **1.47** | **51** | 10797527 | growth arrest and DNA-damage-inducible, gamma |
| Hdac1 | NM_001025409 | **0.74** | **-127** | 10880012 | histone deacetylase 1 |
| Helq | NM_001014134 | **0.73** | **-15** | 10771371 | helicase, POLQ-like |
| Hells | NM_001106371 | **1.92** | **104** | 10715200 | helicase, lymphoid specific |
| Hist1h2bn | NM_001106114 | **0.75** | **-99** | 10798494 | histone cluster 1, H2bn |
| Jmjd1c | XP_228122 | **1.31** | **43** | 10832808 | jumonji domain containing 1C |
| L3mbtl3 | NM_001107612 | **1.45** | **36** | 10702165 | l(3)mbt-like 3 (Drosophila) |
| LOC682649 | ENSRNOT0000004[illegible] | **0.76** | **-278** | 10795265 | similar to Histone H2A type 1 |
| Recql | NM_001012098 | **0.81** | **-37** | 10866672 | RecQ protein-like (DNA helicase Q1-like) |
| LOC100361110 | XM_002727043 | **0.81** | **-12** | 10917992 | histone H3.3B-like |
| RGD1564767 | NM_001024282 | **0.76** | **-44** | 10795196 | Histone H2a |
| **Golgi Apparatus** | | | | | |
| Ap3m1 | NM_133593 | **1.26** | **96** | 10779018 | adaptor-related protein complex 3, mu 1 subunit |
| Ap3m2 | NM_133305 | **0.68** | **-17** | 10789074 | adaptor-related protein complex 3, mu 2 subunit |
| Ap4b1 | NM_001107709 | **0.79** | **-44** | 10818021 | adaptor-related protein complex 4, beta 1 subunit |
| Gbf1 | XM_219953 | **0.76** | **-125** | 10715746 | golgi-specific brefeldin A resistant guanine nucleotide exchange factor 1 |
| Golim4 | BC091440 | **1.58** | **154** | 10823742 | golgi integral membrane protein 4 |
| Hs6st1 | NM_001108210 | **1.40** | **100** | 10922489 | heparan sulfate 6-O-sulfotransferase 1 |
| Ogt | NM_017107 | **1.26** | **319** | 10934445 | O-linked N-acetylglucosamine (GlcNAc) transferase (UDP-N-acetylglucosamine:polypeptide-N-acetylglucosaminyl transferase) |
| Plbd2 | NM_139255 | **0.83** | **-93** | 10758830 | phospholipase B domain containing 2 |
| Pofut1 | NM_001002278 | **0.78** | **-18** | 10841011 | protein O-fucosyltransferase 1 |
| Pomt1 | NM_053406 | **0.76** | **-85** | 10835530 | protein-O-mannosyltransferase 1 |
| Rpn2 | NM_031698 | **0.83** | **-215** | 10841602 | ribophorin II |
| RGD1560511 | NM_001107355 | **1.79** | **355** | 10798666 | similar to Vps41 protein |
| **Growth Factors** | | | | | |
| Creld2 | NM_001037208 | **0.80** | **-97** | 10898456 | cysteine-rich with EGF-like domains 2 |
| Hdgfl1 | NM_133549 | **0.52** | **-133** | 10795072 | hepatoma derived growth factor-like 1 |
| Igf1r | NM_052807 | **0.82** | **-53** | 10707889 | insulin-like growth factor 1 receptor |
| Insl6 | NM_022583 | **0.58** | **-15** | 10729596 | insulin-like 6 |
| Sep11 | NM_001107208 | **1.28** | **58** | 10775769 | septin 11 |
| **Immune Response** | | | | | |
| F3 | NM_013057 | **1.86** | **101** | 10818708 | coagulation factor III (thromboplastin, tissue factor) |
| Hmcn1 | XM_222716 | **1.34** | **44** | 10768426 | hemicentin 1 |
| Lair1 | NM_001029928 | **1.34** | **77** | 10704133 | leukocyte-associated immunoglobulin-like receptor 1 |
| Lims1 | NM_001145456 | **1.24** | **84** | 10833523 | LIM and senescent cell antigen-like domains 1 |

| | | | | | |
|---|---|---|---|---|---|
| Stag1 | NM_001108179 | **1.46** | **129** | 10912525 | stromal antigen 1 |
| LOC687600 | XR_006786 | **0.74** | **-31** | 10761917 | similar to B-cell CLL/lymphoma 7A |
| LOC690041 | XP_001073004 | **1.31** | **10** | 10940120 | X-linked lymphocyte-regulated 3A |
| **Metabolism & Transport** | | | | | |
| Acbd5 | NM_001077635 | **1.37** | **168** | 10796785 | acyl-Coenzyme A binding domain containing 5 |
| Arsk | NM_001047917 | **1.44** | **41** | 10820073 | arylsulfatase family, member K |
| Atox1 | NM_053359 | **0.79** | **-16** | 10742813 | ATX1 antioxidant protein 1 homolog (yeast) |
| Atp5e | NM_139099 | **0.76** | **-89** | 10773636 | ATP synthase, H+ transporting, mitochondrial F1 complex, epsilon subunit |
| Atp5i | NM_080481 | **0.67** | **-40** | 10770996 | ATP synthase, H+ transporting, mitochondrial F0 complex, subunit E |
| Atp5o | NM_138883 | **0.81** | **-42** | 10753198 | ATP synthase, H+ transporting, mitochondrial F1 complex, O subunit |
| Atp6v1g1 | NM_001106660 | **0.76** | **-259** | 10869483 | ATPase, H transporting, lysosomal V1 subunit G1 |
| Atp8b3 | XM_234937 | **0.60** | **-93** | 10893797 | ATPase, Class I, type 8B, member 3 |
| Cbr4 | NM_182672 | **0.78** | **-26** | 10791401 | carbonyl reductase 4 |
| Cds1 | NM_031242 | **0.61** | **-62** | 10775503 | CDP-diacylglycerol synthase 1 |
| Cp | NM_012532 | **1.93** | **109** | 10814430 | ceruloplasmin |
| Cpt2 | NM_012930 | **0.60** | **-94** | 10878564 | carnitine palmitoyltransferase 2 |
| Dis3 | NM_001127483 | **1.51** | **135** | 10785565 | DIS3 mitotic control homolog (S. cerevisiae) |
| Dlat | NM_031025 | **0.74** | **-59** | 10793304 | dihydrolipoamide S-acetyltransferase |
| Dnttip1 | NM_134400 | **0.81** | **-33** | 10842172 | deoxynucleotidyltransferase, terminal, interacting protein 1 |
| Exoc3l | NM_001106178 | **1.31** | **132** | 10810570 | exocyst complex component 3-like |
| Exosc5 | NM_001107493 | **0.80** | **-74** | 10705202 | exosome component 5 |
| Faah | NM_024132 | **0.74** | **-16** | 10871182 | fatty acid amide hydrolase |
| Gpat2 | NM_001168529 | **0.71** | **-28** | 10839598 | glycerol-3-phosphate acyltransferase 2, mitochondrial |
| Gpd2 | NM_012736 | **1.38** | **20** | 10836277 | glycerol-3-phosphate dehydrogenase 2, mitochondrial |
| Hadhb | NM_133618 | **0.81** | **-128** | 10889074 | hydroxyacyl-Coenzyme A dehydrogenase/3-ketoacyl-Coenzyme A thiolase/enoyl-Coenzyme A hydratase (trifunctional protein), beta subunit |
| Hagh | NM_033349 | **0.72** | **-71** | 10732228 | hydroxyacyl glutathione hydrolase |
| Ldhal6b | NM_183334 | **0.72** | **-14** | 10717861 | lactate dehydrogenase A-like 6B |
| Lhpp | NM_001009706 | **0.83** | **-30** | 10711715 | phospholysine phosphohistidine inorganic pyrophosphate phosphatase |
| Mdh1b | XP_237203 | **1.58** | **305** | 10928522 | malate dehydrogenase 1B, NAD (soluble) |
| Ndufa12 | NM_001106781 | **0.72** | **-135** | 10894993 | NADH dehydrogenase (ubiquinone) 1 alpha subcomplex, 12 |
| Ndufb5 | NM_001106426 | **0.81** | **-177** | 10814788 | NADH dehydrogenase (ubiquinone) 1 beta subcomplex, 5 |
| Ndufb9 | NM_001127294 | **0.74** | **-74** | 10903936 | NADH dehydrogenase (ubiquinone) 1 beta subcomplex, 9 |
| Ndufs5 | NM_001030052 | **0.66** | **-51** | 10879662 | NADH dehydrogenase (ubiquinone) Fe-S protein 5 |
| Ndufs6 | NM_019223 | **0.71** | **-87** | 10793236 | NADH dehydrogenase (ubiquinone) Fe-S protein 6 |
| Nppc | NM_053750 | **1.39** | **16** | 10929606 | natriuretic peptide precursor C |
| Nup205 | NM_001108620 | **0.77** | **-53** | 10854494 | nucleoporin 205 |
| Oscp1 | NM_001029923 | **0.59** | **-67** | 10871984 | organic solute carrier partner 1 |
| Pdhb | NM_001007620 | **0.83** | **-156** | 10779485 | pyruvate dehydrogenase (lipoamide) beta |
| Pdia6 | NM_001004442 | **0.79** | **-189** | 10892859 | protein disulfide isomerase family A, member 6 |
| Pggt1b | NM_031082 | **1.53** | **119** | 10804361 | protein geranylgeranyltransferase type I, beta subunit |
| Pgp | NM_001169152 | **0.81** | **-99** | 10732076 | phosphoglycolate phosphatase |
| Pigw | NM_194461 | **1.29** | **45** | 10745782 | phosphatidylinositol glycan anchor biosynthesis, class W |
| Pla2g6 | NM_001005560 | **0.80** | **-20** | 10905406 | phospholipase A2, group VI (cytosolic, calcium-independent) |
| Plcl1 | NM_053456 | **1.39** | **15** | 10923361 | phospholipase C-like 1 |
| Pnpla2 | NM_001108509 | **0.82** | **-45** | 10712317 | patatin-like phospholipase domain containing 2 |
| Ppih | XM_345576 | **0.83** | **-55** | 10871582 | peptidylprolyl isomerase H (cyclophilin H) |
| Ppwd1 | NM_001106406 | **1.73** | **87** | 10821149 | peptidylprolyl isomerase domain and WD repeat containing 1 |
| RGD1306402 | BC093611 | **1.21** | **43** | 10774577 | pseudouridylate synthase 10 |
| Qsox2 | NM_001109434 | **0.74** | **-68** | 10843664 | quiescin Q6 sulfhydryl oxidase 2 |
| Slc25a36 | XP_576451 | **1.33** | **428** | 10919433 | solute carrier family 25, member 36 |
| Slc25a40 | NM_001037186 | **1.32** | **36** | 10853292 | solute carrier family 25, member 40 |
| Slc26a5 | NM_030840 | **0.57** | **-21** | 10853091 | solute carrier family 26, member 5 (prestin) |
| Slc38a7 | NM_001003705 | **0.82** | **-17** | 10805802 | solute carrier family 38, member 7 |
| Thnsl1 | NM_001025035 | **0.71** | **-19** | 10796696 | threonine synthase-like 1 (S. cerevisiae) |
| Tmed5 | NM_001007619 | **1.45** | **21** | 10771004 | transmembrane emp24 protein transport domain containing 5 |
| Tomm7 | NM_001135174 | **0.72** | **-264** | 10852964 | translocase of outer mitochondrial membrane 7 homolog (yeast) |
| Trappc4 | NM_001003708 | **0.71** | **-97** | 10916785 | trafficking protein particle complex 4 |

| | | | | | |
|---|---|---|---|---|---|
| Ugp2 | NM_001024743 | **1.26** | **114** | 10778647 | UDP-glucose pyrophosphorylase 2 |
| Xpot | NM_001108102 | **0.77** | **-141** | 10902735 | exportin, tRNA (nuclear export receptor for tRNAs) |
| LOC687295 | XM_001065015 | **0.77** | **-60** | 10720161 | similar to translocase of inner mitochondrial membrane 50 homolog |
| LOC685431 | XR_086366 | **1.21** | **82** | 10937570 | similar to Alpha-enolase (2-phospho-D-glycerate hydro-lyase) (Non-neural enolase) (NNE) (Enolase 1) |
| **Proteolysis** | | | | | |
| Adam10 | Z48444 | **1.48** | **278** | 10918545 | ADAM metallopeptidase domain 10 |
| Dcun1d1 | NM_001107668 | **1.43** | **184** | 10822792 | DCN1, defective in cullin neddylation 1, domain containing 1 (S. cerevisiae) |
| Prss47 | XM_001056923 | **0.78** | **-11** | 10793409 | protease, serine, 47 |
| Mbtps2 | BC097391 | **1.21** | **39** | 10933634 | membrane-bound transcription factor peptidase, site 2 |
| Pcolce | NM_019237 | **1.27** | **15** | 10760971 | procollagen C-endopeptidase enhancer |
| Psmb4 | NM_031629 | **0.78** | **-266** | 10824884 | proteasome (prosome, macropain) subunit, beta type 4 |
| Psmb6 | NM_057099 | **0.77** | **-44** | 10735244 | proteasome (prosome, macropain) subunit, beta type 6 |
| Psmd12 | NM_001005875 | **0.78** | **-117** | 10739225 | proteasome (prosome, macropain) 26S subunit, non-ATPase, 12 |
| Psmd6 | NM_198730 | **0.80** | **-154** | 10779357 | proteasome (prosome, macropain) 26S subunit, non-ATPase, 6 |
| Rbx1 | NM_001034135 | **0.79** | **-122** | 10897912 | ring-box 1 |
| Rnf111 | NM_001106836 | **1.25** | **56** | 10918519 | ring finger protein 111 |
| Rnf146 | BC083675 | **0.83** | **-21** | 10702428 | ring finger protein 146 |
| Rnft1 | XM_340875 | **1.24** | **68** | 10737380 | ring finger protein, transmembrane 1 |
| Uqcrq | NM_001025134 | **0.76** | **-64** | 10742601 | ubiquinol-cytochrome c reductase, complex III subunit VII |
| Usp33 | BC092624 | **1.39** | **186** | 10819790 | ubiquitin specific peptidase 33 |
| Vps45 | NM_172072 | **0.75** | **-118** | 10825120 | vacuolar protein sorting 45 homolog (S. cerevisiae) |
| **Receptors & Binding Proteins** | | | | | |
| Abce1 | NM_001108446 | **1.27** | **101** | 10806960 | ATP-binding cassette, sub-family E (OABP), member 1 |
| Efcab10 | NM_001108706 | **0.68** | **-17** | 10884200 | EF-hand calcium binding domain 10 |
| Il13ra1 | NM_145789 | **1.54** | **19** | 10936365 | interleukin 13 receptor, alpha 1 |
| Ireb2 | NM_022863 | **1.32** | **126** | 10910094 | iron responsive element binding protein 2 |
| M6prbp1 | XM_001061015 | **1.27** | **25** | 10930984 | mannose-6-phosphate receptor binding protein 1 |
| Olr769 | NM_001000371 | **1.26** | **13** | 10848113 | olfactory receptor 769 |
| Oprs1 | NM_030996 | **0.77** | **-33** | 10876185 | sigma non-opioid intracellular receptor 1 |
| Plxdc2 | NM_001108422 | **1.56** | **16** | 10796564 | plexin domain containing 2 |
| Rora | NM_001106834 | **1.37** | **40** | 10911250 | RAR-related orphan receptor alpha |
| Sec61a2 | NM_001170343 | **0.73** | **-357** | 10796280 | Sec61 alpha 2 subunit (S. cerevisiae) |
| Stxbp6 | XM_343057 | **1.38** | **15** | 10889772 | syntaxin binding protein 6 (amisyn) |
| **Signaling** | | | | | |
| Ak7 | NM_001108055 | **0.75** | **-19** | 10886652 | adenylate kinase 7 |
| Alcam | NM_031753 | **1.94** | **110** | 10750878 | activated leukocyte cell adhesion molecule |
| Aurkc | NM_001106221 | **1.23** | **13** | 10718658 | aurora kinase C |
| Calm3 | NM_012518 | **0.73** | **-459** | 10719294 | calmodulin 3 |
| Cdc42ep3 | NM_001048044 | **0.74** | **-50** | 10887939 | CDC42 effector protein (Rho GTPase binding) 3 |
| Clk1 | NM_001106913 | **1.39** | **501** | 10928229 | CDC-like kinase 1 |
| Cyr61 | NM_031327 | **1.53** | **204** | 10827231 | cysteine-rich, angiogenic inducer, 61 |
| Dennd2c | XM_001067655 | **1.30** | **25** | 10817959 | DENN/MADD domain containing 2C |
| Drg1 | NM_001009685 | **0.72** | **-270** | 10777918 | developmentally regulated GTP binding protein 1 |
| Dusp10 | NM_001105734 | **1.57** | **11** | 10766463 | dual specificity phosphatase 10 |
| Efhd2 | NM_001031648 | **1.24** | **225** | 10881211 | EF-hand domain family, member D2 |
| Ei24 | NM_001025660 | **0.77** | **-118** | 10916144 | etoposide induced 2.4 mRNA |
| Elmod3 | NM_001013087 | **0.75** | **-20** | 10863246 | ELMO/CED-12 domain containing 3 |
| Erbb4 | NM_021687 | **1.41** | **92** | 10928636 | v-erb-a erythroblastic leukemia viral oncogene homolog 4 (avian) |
| Fastkd2 | NM_001009673 | **1.21** | **46** | 10923904 | FAST kinase domains 2 |
| Fgd4 | NM_139263 | **1.36** | **32** | 10755875 | FYVE, RhoGEF and PH domain containing 4 |
| Fkbp15 | XM_342846 | **1.25** | **32** | 10877380 | FK506 binding protein 15 |
| Gtpbp1 | NM_001130496 | **0.83** | **-87** | 10897709 | GTP binding protein 1 |
| Hsp90aa1 | NM_175761 | **1.56** | **2165** | 10892184 | heat shock protein 90, alpha (cytosolic), class A member 1 |
| Jak2 | NM_031514 | **1.38** | **93** | 10714667 | Janus kinase 2 |
| Map2k4 | NM_001030023 | **1.21** | **162** | 10743668 | mitogen activated protein kinase kinase 4 |
| Mapk7 | AJ005424 | **0.82** | **-46** | 10743427 | mitogen-activated protein kinase 7 |
| Mapk8ip2 | BC105884 | **0.62** | **-22** | 10898606 | mitogen-activated protein kinase 8 interacting protein 2 |
| Narg2 | XM_001075498 | **1.48** | **49** | 10911270 | NMDA receptor regulated 2 |

| | | | | | |
|---|---|---|---|---|---|
| Nek1 | NM_001106082 | **1.37** | **63** | 10791421 | NIMA (never in mitosis gene a)-related expressed kinase 1 |
| Pde1a | NM_030871 | **1.46** | **12** | 10846694 | phosphodiesterase 1A, calmodulin-dependent |
| Pih1d2 | XP_236221 | **1.50** | **30** | 10909886 | PIH1 domain containing 2 |
| Pik3ca | NM_133399 | **1.22** | **53** | 10814726 | phosphoinositide-3-kinase, catalytic, alpha polypeptide |
| Pik3r3 | NM_022213 | **0.82** | **-42** | 10871229 | phosphoinositide-3-kinase, regulatory subunit 3 (gamma) |
| Pim1 | NM_017034 | **1.31** | **121** | 10828884 | pim-1 oncogene |
| Plekha8 | NM_001109235 | **0.74** | **-60** | 10855650 | pleckstrin homology domain containing, family A (phosphoinositide binding specific) member 8 |
| Pmaip1 | NM_001008385 | **1.39** | **16** | 10802375 | phorbol-12-myristate-13-acetate-induced protein 1 |
| Ppp1r11 | NM_212542 | **0.78** | **-21** | 10827701 | protein phosphatase 1, regulatory (inhibitor) subunit 11 |
| Prkaa2 | NM_023991 | **1.21** | **28** | 10878416 | protein kinase, AMP-activated, alpha 2 catalytic subunit |
| Prkdc | NM_001108327 | **1.46** | **73** | 10755897 | protein kinase, DNA activated, catalytic polypeptide |
| Ptprg | NM_134356 | **1.26** | **18** | 10782609 | protein tyrosine phosphatase, receptor type, G |
| Purg | XM_001068166 | **1.42** | **12** | 10792101 | purine-rich element binding protein G |
| Radil | NM_001037218 | **0.68** | **-22** | 10756853 | Ras association and DIL domains |
| Ranbp1 | NM_001108324 | **0.83** | **-104** | 10751448 | RAN binding protein 1 |
| Rasl2-9 | NM_001166675 | **0.80** | **-19** | 10703905 | RAS-like, family 2, locus 9 |
| Riok2 | NM_001009687 | **1.45** | **166** | 10703403 | RIO kinase 2 (yeast) |
| Rock2 | NM_013022 | **1.73** | **128** | 10883726 | Rho-associated coiled-coil containing protein kinase 2 |
| Sipa1l1 | NM_139330 | **0.66** | **-63** | 10885740 | signal-induced proliferation-associated 1 like 1 |
| Stk36 | NM_001173986 | **0.82** | **-17** | 10924363 | serine/threonine kinase 36 (fused homolog, Drosophila) |
| Tbc1d1 | XM_341215 | **0.75** | **-68** | 10776915 | TBC1 domain family, member 1 |
| Tec | NM_053432 | **1.28** | **10** | 10772408 | tec protein tyrosine kinase |
| RGD1560527 | XM_001055752 | **0.73** | **-23** | 10797590 | similar to serine/threonine kinase |
| RGD1565493 | XM_002729803 | **1.40** | **53** | 10903286 | similar to DKFZP434I092 protein |
| **Transcription** | | | | | |
| Armcx3 | NM_001014273 | **1.30** | **213** | 10934967 | armadillo repeat containing, X-linked 3 |
| Atf2 | NM_031018 | **1.26** | **270** | 10846233 | activating transcription factor 2 |
| Bin3 | NM_001013186 | **0.78** | **-61** | 10781344 | bridging integrator 3 |
| Brms1 | NM_001009605 | **0.80** | **-91** | 10712978 | breast cancer metastasis-suppressor 1 |
| C1d | NM_001106021 | **1.31** | **72** | 10774331 | nuclear DNA binding protein |
| Casc5 | NM_001170594 | **1.28** | **45** | 10838733 | cancer susceptibility candidate 5 |
| Ccdc42 | NM_001107009 | **0.59** | **-33** | 10734761 | coiled-coil domain containing 42 |
| Ccdc56 | NM_001109047 | **0.80** | **-32** | 10747617 | coiled-coil domain containing 56 |
| Ccdc59 | NM_001108090 | **1.37** | **34** | 10895268 | coiled-coil domain containing 59 |
| Ccdc82 | NM_001007660 | **1.44** | **42** | 10907992 | coiled-coil domain containing 82 |
| Cebpz | NM_001108701 | **1.48** | **228** | 10887901 | CCAAT/enhancer binding protein zeta |
| Cnot3 | NM_001107471 | **0.82** | **-40** | 10718520 | CCR4-NOT transcription complex, subunit 3 |
| Dhx15 | XM_214053 | **1.26** | **271** | 10772967 | DEAH (Asp-Glu-Ala-His) box polypeptide 15 |
| Dnaja1 | NM_022934 | **1.65** | **1058** | 10868289 | DnaJ (Hsp40) homolog, subfamily A, member 1 |
| E2f5 | U31668 | **0.65** | **-47** | 10718609 | E2F transcription factor 5 |
| Elp2 | NM_001034145 | **0.81** | **-147** | 10800522 | elongation protein 2 homolog (S. cerevisiae) |
| Hif1a | NM_024359 | **1.53** | **324** | 10885251 | hypoxia-inducible factor 1, alpha subunit (basic helix-loop-helix transcription factor) |
| Htatsf1 | NM_001108259 | **1.69** | **99** | 10935589 | HIV-1 Tat specific factor 1 |
| Junb | NM_021836 | **1.23** | **161** | 10806585 | jun B proto-oncogene |
| Klf4 | NM_053713 | **1.48** | **123** | 10876838 | Kruppel-like factor 4 (gut) |
| Klf5 | NM_053394 | **1.48** | **17** | 10781829 | Kruppel-like factor 5 |
| Klhl9 | NM_001107944 | **1.44** | **340** | 10877984 | kelch-like 9 (Drosophila) |
| Luzp1 | AF181259 | **1.57** | **33** | 10873012 | leucine zipper protein 1 |
| Mdm4 | NM_001012026 | **1.20** | **182** | 10767663 | Mdm4 p53 binding protein homolog (mouse) |
| Med11 | NM_001105799 | **0.73** | **-12** | 10735217 | mediator complex subunit 11 |
| Med13 | NM_001107035 | **1.27** | **168** | 10745897 | mediator complex subunit 13 |
| Mpp5 | NM_001108034 | **1.45** | **109** | 10885500 | membrane protein, palmitoylated 5 (MAGUK p55 subfamily member 5) |
| Mum1 | NM_001108736 | **0.71** | **-50** | 10900554 | melanoma associated antigen (mutated) 1 |
| Nol10 | NM_001014076 | **0.82** | **-26** | 10883763 | nucleolar protein 10 |
| Npat | NM_001108147 | **1.52** | **76** | 10910015 | nuclear protein, ataxia-telangiectasia locus |
| Nsbp1 | NM_001134706 | **1.46** | **255** | 10707658 | nucleosomal binding protein 1 |
| Plag1 | NM_001008316 | **1.55** | **11** | 10875282 | pleiomorphic adenoma gene 1 |
| Pole3 | NM_001007652 | **0.76** | **-99** | 10905186 | polymerase (DNA directed), epsilon 3 (p17 subunit) |

| | | | | | |
|---|---|---|---|---|---|
| Polr2l-ps1 | XM_577644 | **0.74** | **-50** | 10809086 | polymerase (RNA) II (DNA directed) polypeptide L, pseudogene 1 |
| Rfc3 | NM_001009629 | **0.83** | **-22** | 10759752 | replication factor C (activator 1) 3 |
| Rogdi | NM_001024864 | **0.83** | **-24** | 10731686 | rogdi homolog (Drosophila) |
| Sarnp | NM_001033070 | **1.31** | **276** | 10893247 | SAP domain containing ribonucleoprotein |
| Taf1d | NM_001014207 | **1.24** | **52** | 10908089 | TATA box binding protein (TBP)-associated factor, RNA polymerase I, D, 41kDa |
| Tbp | NM_001004198 | **0.80** | **-83** | 10703364 | TATA box binding protein |
| Tcea2 | NM_057098 | **0.67** | **-108** | 10843072 | transcription elongation factor A (SII), 2 |
| Ybx2 | NC_005109 | **0.65** | **-169** | 10735077 | Y box binding protein 2 |
| Zbtb10 | NM_024489 | **1.27** | **151** | 10822330 | zinc finger and BTB domain containing 10 |
| Zfp157 | XM_001057321 | **1.74** | **70** | 10760768 | zinc finger protein 157 |
| Zfp187 | XM_577579 | **1.65** | **109** | 10795319 | zinc finger protein 187 |
| Zfp217 | NM_001107813 | **0.75** | **-51** | 10852050 | zinc finger protein 217 |
| Zfp317 | NM_001134634 | **1.42** | **27** | 10908182 | zinc finger protein 317 |
| Zfp322a | NM_001135084 | **1.89** | **83** | 10795258 | zinc finger protein 322a |
| Zfp330 | NM_001108443 | **1.20** | **11** | 10806886 | zinc finger protein 330 |
| Zfp347 | AB047637 | **1.51** | **15** | 10893431 | zinc finger protein 347 |
| Zfx | NM_001109017 | **1.73** | **139** | 10938396 | zinc finger protein X-linked |
| Zmat2 | NM_001135582 | **0.78** | **-118** | 10801129 | zinc finger, matrin type 2 |
| Znhit1 | XM_001076915 | **0.68** | **-34** | 10761037 | zinc finger, HIT domain containing 1 |
| Znhit2 | NM_001107574 | **0.63** | **-61** | 10713184 | zinc finger, HIT type 2 |
| Crebzf | NM_001106279 | **1.23** | **107** | 10708616 | CREB/ATF bZIP transcription factor |
| Tceanc | NM_001109015 | **1.21** | **11** | 10933362 | transcription elongation factor A (SII) N-terminal and central domain containing |
| LOC100125368 | NM_001139487 | **1.42** | **54** | 10900136 | zinc finger protein LOC100125368 |
| LOC685925 | XM_002725924 | **1.22** | **16** | 10814162 | zinc finger protein 455-like |
| RGD1564061 | NM_001135782 | **0.70** | **-72** | 10873801 | PRAME family member 8 |
| RGD1308290 | NM_001047902 | **1.34** | **144** | 10817202 | zinc finger CCCH-type containing 11A |
| RGD1564807 | XM_225049 | **0.71** | **-11** | 10793060 | similar to zinc finger protein, subfamily 1A, 5 |
| **Translation & Protein Modification** | | | | | |
| Arl3 | NM_022700 | **0.68** | **-172** | 10730591 | ADP-ribosylation factor-like 3 |
| Cct7 | NM_001106603 | **0.75** | **-176** | 10856884 | chaperonin containing Tcp1, subunit 7 (eta) |
| Cwc22 | NM_001047959 | **1.43** | **45** | 10885608 | CWC22 spliceosome-associated protein homolog (S. cerevisiae) |
| Dcp2 | NM_001170469 | **1.22** | **36** | 10801494 | DCP2 decapping enzyme homolog (S. cerevisiae) |
| Eef2k | NM_012947 | **1.25** | **13** | 10710494 | eukaryotic elongation factor-2 kinase |
| Eif1ay | NM_001106963 | **1.30** | **49** | 10938019 | eukaryotic translation initiation factor 1A, Y-linked |
| Eif2b2 | NM_032058 | **0.73** | **-43** | 10886014 | eukaryotic translation initiation factor 2B, subunit 2 beta |
| Eif2s2 | NM_199380 | **1.26** | **97** | 10850970 | eukaryotic translation initiation factor 2, subunit 2 beta |
| Eif5a | NM_001033681 | **0.82** | **-663** | 10744318 | eukaryotic translation initiation factor 5A |
| Eif5a2 | NM_001100697 | **1.30** | **34** | 10814644 | eukaryotic translation initiation factor 5A2 |
| Eif5b | NM_001110141 | **1.56** | **147** | 10703648 | eukaryotic translation initiation factor 5B |
| Hnrnpa2b1 | NM_001104613 | **1.27** | **223** | 10862522 | heterogeneous nuclear ribonucleoprotein A2/B1 |
| Lsm7 | NM_001108732 | **0.71** | **-27** | 10893684 | LSM7 homolog, U6 small nuclear RNA associated (S. cerevisiae) |
| Mrpl22 | NM_001105781 | **0.79** | **-16** | 10733782 | mitochondrial ribosomal protein L22 |
| Mrpl41 | NM_001013426 | **0.78** | **-29** | 10911993 | mitochondrial ribosomal protein L41 |
| Mrpl54 | NM_001106770 | **0.75** | **-130** | 10893586 | mitochondrial ribosomal protein L54 |
| Mrps18c | NM_001105996 | **0.67** | **-270** | 10775532 | mitochondrial ribosomal protein S18C |
| Phax | NM_173133 | **0.79** | **-22** | 10801810 | phosphorylated adaptor for RNA export |
| Prpf8 | BC099197 | **0.82** | **-146** | 10735897 | PRP8 pre-mRNA processing factor 8 homolog (S. cerevisiae) |
| Rbm17 | NM_001013058 | **0.83** | **-184** | 10796134 | RNA binding motif protein 17 |
| Rpl27 | NM_022514 | **0.82** | **-105** | 10738409 | ribosomal protein L27 |
| Rps4x | NM_001007600 | **1.22** | **362** | 10938722 | ribosomal protein S4, X-linked |
| Rps4y2 | NM_001109612 | **0.77** | **-73** | 10859760 | ribosomal protein S4, Y-linked 2 |
| Rpusd2 | NM_001135845 | **0.77** | **-10** | 10838729 | RNA pseudouridylate synthase domain containing 2 |
| Srrm2 | XP_220207 | **1.22** | **224** | 10731980 | serine/arginine repetitive matrix 2 |
| Taf5l | NM_001107442 | **0.81** | **-18** | 10703465 | TAF5-like RNA polymerase II, p300/CBP-associated factor (PCAF)-associated factor |
| Thoc7 | XM_001069423 | **0.70** | **-55** | 10779368 | THO complex 7 homolog (Drosophila) |
| Trove2 | NM_001107183 | **1.20** | **24** | 10768323 | TROVE domain family, member 2 |
| RGD1560949 | XM_001078650 | **0.73** | **-22** | 10808338 | similar to testis nuclear RNA-binding protein-like |

| | | | | | |
|---|---|---|---|---|---|
| LOC681258 | ENSRNOT0000005! | **1.26** | **137** | 10899204 | similar to La-related protein 4 (La ribonucleoprotein domain family member 4) |
| **Miscellaneous & Unknown** | | | | | |
| Bxdc2 | NM_001029915 | **1.39** | **90** | 10821914 | brix domain containing 2 |
| Dph3 | NM_001134850 | **0.71** | **-77** | 10790305 | DPH3, KTI11 homolog (S. cerevisiae) |
| Fam164a | BC168976 | **1.36** | **23** | 10822377 | family with sequence similarity 164, member A |
| Fam169a | XM_226706 | **1.32** | **196** | 10812656 | family with sequence similarity 169, member A |
| Hiatl1 | NM_001107334 | **1.26** | **50** | 10793397 | hippocampus abundant transcript-like 1 |
| Ift122 | BC087667 | **0.76** | **-80** | 10858038 | intraflagellar transport 122 homolog (Chlamydomonas) |
| Ift57 | NM_001107093 | **0.72** | **-85** | 10753784 | intraflagellar transport 57 homolog (Chlamydomonas) |
| Lsmd1 | NM_001105794 | **0.78** | **-47** | 10708152 | LSM domain containing 1 |
| Nipsnap3a | NM_001009422 | **1.33** | **13** | 10869094 | nipsnap homolog 3B (C. elegans) |
| Otud1 | XM_574086 | **0.80** | **-13** | 10796673 | OTU domain containing 1 |
| Tmem134 | NM_001078647 | **0.80** | **-84** | 10712816 | transmembrane protein 134 |
| Tmem9 | NM_001105953 | **0.78** | **-23** | 10764260 | transmembrane protein 9 |
| Trim52 | NM_001106056 | **0.83** | **-50** | 10781956 | tripartite motif-containing 52 |
| Wdr31 | NM_001011976 | **0.67** | **-91** | 10877418 | WD repeat domain 31 |
| Wdr35l | NM_001099340 | **0.77** | **-130** | 10883557 | WD repeat domain 35-like |
| Wdr45 | NM_001013958 | **1.27** | **28** | 10937064 | WD repeat domain 45 |
| Wdr51a | NM_001109296 | **0.80** | **-12** | 10912826 | WD repeat domain 51A |
| Wdr53 | NM_001109055 | **0.75** | **-11** | 10754862 | WD repeat domain 53 |
| Wdr66 | XM_222180 | **0.59** | **-35** | 10761921 | WD repeat domain 66 |
| Dip2c | NM_001107360 | **0.81** | **-45** | 10799158 | DIP2 disco-interacting protein 2 homolog C (Drosophila) |
| LOC362855 | BC078924 | **0.80** | **-67** | 10895581 | P55 |
| Ptges3l1 | BC087125 | **1.65** | **20** | 10938126 | prostaglandin E synthase 3-like 1 |
| Rsrc2 | NM_001014128 | **1.51** | **143** | 10758354 | arginine/serine-rich coiled-coil 2 |
| Tmem107 | NM_001109648 | **0.59** | **-66** | 10734866 | transmembrane protein 107 |
| Tmem181 | BC168231 | **0.78** | **-41** | 10702880 | transmembrane protein 181 |
| LOC500034 | NM_001109221 | **1.40** | **132** | 10861117 | similar to CG3570-PA |
| LOC680262 | XM_001056376 | **0.64** | **-45** | 10916073 | hypothetical protein LOC680262 |
| LOC686050 | XM_001066348 | **1.27** | **42** | 10877141 | hypothetical protein LOC686050 |
| LOC686661 | XM_001075174 | **1.32** | **13** | 10742868 | similar to CG1998-PA |
| LOC688211 | XM_001062937 | **1.24** | **70** | 10747891 | hypothetical protein LOC685233 |
| RGD1563106 | NM_001107010 | **0.80** | **-128** | 10734830 | similar to novel protein |
| RGD1563422 | NM_001077643 | **0.73** | **-54** | 10765293 | similar to Brain protein 44 |
| RGD1305899 | XM_001058977 | **0.60** | **-102** | 10852385 | similar to Protein C20orf158 |
| RGD1307051 | NM_001108091 | **1.65** | **27** | 10902112 | similar to hypothetical protein FLJ21963 |
| RGD1309540 | NM_001013918 | **0.77** | **-69** | 10847375 | similar to hypothetical protein MGC40841; similar to hypothetical protein MGC4707 |
| RGD1309995 | XM_235003 | **1.22** | **67** | 10901367 | similar to CG13957-PA |
| RGD1559496 | NM_001106199 | **0.69** | **-164** | 10808874 | similar to hypothetical protein |
| **EST's** | | | | | |
| LOC100302465 | BC167765 | **0.71** | **-18** | 10726394 | hypothetical LOC100302465 |
| RGD1306936 | NM_001106593 | **0.82** | **-47** | 10855462 | similar to chromosome 7 open reading frame 30 |
| RGD1307947 | NM_001014083 | **1.39** | **21** | 10902039 | similar to RIKEN cDNA C430008C19 |
| RGD1564093 | NM_001105946 | **0.82** | **-134** | 10809993 | similar to RIKEN cDNA 2310036O22 |
| RGD1564792 | XM_580041 | **1.29** | **17** | 10853002 | RGD1564792 |
| RGD1565975 | XM_001065138 | **0.75** | **-32** | 10806246 | RGD1565975 |
| | BC089062 | **1.64** | **31** | 10877751 | |
| | BC090353 | **1.63** | **1134** | 10728028 | |
| | ENSRNOT0000000( | **0.77** | **-14** | 10926153 | |
| | ENSRNOT0000000: | **0.67** | **-11** | 10759597 | |
| | ENSRNOT0000000: | **1.47** | **56** | 10935456 | |
| | ENSRNOT0000001( | **0.64** | **-25** | 10849614 | |
| | ENSRNOT0000002( | **1.38** | **57** | 10846151 | |
| | ENSRNOT0000002' | **0.71** | **-16** | 10706637 | |
| | ENSRNOT0000002: | **0.75** | **-58** | 10713084 | |
| | ENSRNOT0000003( | **0.74** | **-24** | 10840394 | |
| | ENSRNOT0000003: | **1.39** | **40** | 10937839 | |
| | ENSRNOT0000003: | **1.33** | **265** | 10928916 | |

| | | | | | | |
|---|---|---|---|---|---|---|
| | ENSRNOT0000004 | **1.20** | **16** | 10804402 | | |
| | ENSRNOT0000004 | **0.78** | **-24** | 10924505 | | |
| | ENSRNOT0000004 | **1.46** | **66** | 10812903 | | |
| | ENSRNOT0000004 | **1.58** | **41** | 10731222 | | |
| | ENSRNOT0000005 | **1.70** | **154** | 10939958 | | |
| | ENSRNOT0000005 | **1.35** | **23** | 10935150 | | |
| | ENSRNOT0000005 | **1.21** | **34** | 10910766 | | |
| | ENSRNOT0000005 | **0.55** | **-134** | 10801135 | | |
| | ENSRNOT0000005 | **1.25** | **15** | 10829163 | | |
| | ENSRNOT0000005 | **1.34** | **14** | 10744141 | | |
| | ENSRNOT0000005 | **1.22** | **18** | 10834600 | | |
| | ENSRNOT0000005 | **0.78** | **-19** | 10702714 | | |
| | ENSRNOT0000005 | **0.78** | **-19** | 10915105 | | |
| | ENSRNOT0000005 | **0.78** | **-45** | 10842742 | | |
| | ENSRNOT0000005 | **1.27** | **80** | 10824548 | | |
| | ENSRNOT0000005 | **0.72** | **-59** | 10938887 | | |
| | ENSRNOT0000006 | **1.57** | **97** | 10775624 | | |
| | ENSRNOT0000006 | **1.37** | **25** | 10869549 | | |
| | ENSRNOT0000007 | **1.30** | **40** | 10834604 | | |
| | ENSRNOT0000007 | **0.73** | **-15** | 10934821 | | |
| | FQ222240 | **1.58** | **85** | 10714078 | | |
| | FQ222682 | **0.77** | **-82** | 10722718 | | |
| | FQ224515 | **1.50** | **46** | 10823593 | | |
| | GENSCAN0000001 | **1.34** | **15** | 10875696 | | |
| | GENSCAN0000003 | **0.65** | **-49** | 10807923 | | |
| | NC_001665 | **0.82** | **-30** | 10930608 | | |
| | NR_031856 | **1.83** | **59** | 10749975 | | |
| | --- | **1.29** | **13** | 10866408 | | |
| | --- | **0.80** | **-11** | 10872885 | | |
| | --- | **0.80** | **-32** | 10864838 | | |
| | --- | **0.63** | **-15** | 10921621 | | |
| | --- | **0.77** | **-13** | 10755627 | | |
| | --- | **1.27** | **38** | 10905770 | | |
| | --- | **1.65** | **22** | 10791419 | | |
| | --- | **1.65** | **31** | 10911847 | | |
