# Supplementary material for: Environmentally Induced Epigenetic Transgenerational Inheritance of Altered Sertoli Cell Transcriptome and Epigenome: Molecular Etiology of Male Infertility
Source: PLoS One. 2013 Mar 28;8(3):e59922. doi: 10.1371/journal.pone.0059922 (PMC3610698; doi:10.1371/journal.pone.0059922)
Supplement: Table S2 — Differential DNA methylation regions (DMR) in F3 generation vinclozolin lineage Sertoli cells. (PDF) [file pone.0059922.s005.pdf]

Supplemental Table S2. Differential DNA methylation regions (DMR) in F3 generation vinclozolin lineage Sertoli cells

Gene symbol	Description	RGD ID	Entrez gene ID	*p* value	Changed region coordinates		
					Chr	Start	End
Abcf3	ATP-binding cassette, subfamily F (GCN20), member 3	1310468	287982	5.12E-12	11	82581404	82582483
Acoxl	Acyl-CoA oxidase-like	1306814	296138	1.44E-16	3	115365654	115366254
Amigo2	Adhesion molecule with Ig like domain 2	727911	300186	7.67E-27	7	135969017	135969617
Angptl4	Angiopoietin-like 4	735058	362850	1.98E-18	7	16267607	16268546
Antxr1	Anthrax toxin receptor 1	1307144	362393	1.80E-30	4	121483647	121484678
Armcx2	Armadillo repeat containing, X-linked 2	1359132	367903	5.74E-35	X	122265349	122266237
Atp6v0b	ATPase, H+ transporting, lysosomal V0 subunit B	1308303	298451	1.92E-09	5	138350342	138351447
Bat2l	Proline-rich coiled-coil 2B	1595906	296637	3.72E-21	3	11290691	11291386
Calca	Calcitonin-related polypeptide alpha	2254	24241	6.29E-13	1	172690139	172691239
Ccdc17	Coiled-coil domain containing 17	1598211	500528	2.36E-17	5	136879913	136881400
Ceacam6	Carcinoembryonic antigen-related cell adhesion molecule 6	1642421	100125369	1.08E-17	1	80107471	80108071
Cox8b	Cytochrome c oxidase, subunit VIIIb	2386	25250	1.38E-16	1	201058346	201059031
Csrnp1	Cysteine-serine-rich nuclear protein 1	1305999	363165	9.95E-10	8	124938760	124939874
Cyp4f5	Cytochrome P450, family 4, subfamily f, polypeptide 5	708364	286905	1.78E-22	7	13119902	13120699
Dctn2	Dynactin 2	1303182	299850	1.05E-23	7	67223573	67224293
Dll3	Delta-like 3 (Drosophila)	70953	114125	1.19E-15	1	83381756	83383501
Dnd1	Dead end homolog 1 (zebrafish)	1583648	679841	1.40E-16	18	29465951	29466771
Ebf3	Early B-cell factor 3	1304956	361668	6.71E-15	1	196931754	196932859
Emilin1	Elastin microfibril interfacer 1	1311209	298845	1.98E-08	6	25440952	25442052
Esrp2	Epithelial splicing regulatory protein 2	1310855	307810	2.19E-13	19	35989148	35990238
Fech	Ferrochelatase	1307556	361338	2.56E-08	18	60708474	60709288
G6b	G6b protein	1303269	406865	2.76E-17	20	3822418	3823018
Gabarapl1	GABA(A) receptor-associated protein like 1	1596143	689161	1.39E-16	4	166802302	166803201
Gad2	Glutamate decarboxylase 2	2653	24380	1.23E-18	17	96258803	96259502
Gas6	Growth arrest specific 6	61913	58935	5.79E-12	16	80896103	80897214
Gene id Chr1_1386.1	Predicted gene	NA	NA	5.55E-23	1	93660805	93661590
Gene id Chr19_655.1	Predicted gene	NA	NA	5.98E-36	19	41441292	41442411
Gene id Chr7_1541.1	Predicted gene	NA	NA	3.26E-25	7	114997310	114998010
Giyd2	SLX1 structure-specific endonuclease subunit homolog B (S. cerevisiae)	1311568	293489	1.00E-31	1	185849341	185850026
Gng8	Guanine nucleotide binding protein (G protein), gamma 8	620808	245986	1.15E-20	1	77218978	77219578
Gp1bb	Glycoprotein Ib (platelet), beta polypeptide	621050	116727	9.67E-10	11	84368047	84368937
Gzmb	Granzyme B	620018	171528	3.87E-08	15	35201955	35202555
Hdc	Histidine decarboxylase	2790	24443	1.02E-15	3	114138885	114139770
Higd2a	HIG1 hypoxia inducible domain family, member 2A	1309691	290999	4.24E-13	17	16084041	16084831
Iffo2	Intermediate filament family orphan 2	1624207	641315	2.52E-11	5	158403134	158403944
Il22ra2	Il22ra2 interleukin 22 receptor, alpha 2	1303169	44986	2.13E-15	1	14875559	14876159
Ilvbl	ilvB (bacterial acetolactate synthase)-like	1307988	362843	1.65E-09	7	12602409	12603404
Isg20l2	Interferon stimulated exonuclease gene 20-like 2	1359413	361977	1.18E-16	2	180008944	180009544
Kb15	Type II keratin Kb15	1565564	366992	9.64E-08	7	140318073	140318965
Kctd11	Potassium channel tetramerisation domain containing 11	1307125	363634	3.37E-13	10	56726062	56726846
Klhl17	Kelch-like 17 (Drosophila)	708444	246757	1.15E-22	5	173066362	173067163
Krt83	Keratin 83	1583565	681126	5.05E-11	7	140185564	140186164
Krt84	Keratin 84	1359402	315320	6.63E-13	7	140287213	140287908
Lhx8	LIM homeobox 8	1308749	365963	6.55E-10	2	252237969	252238667
Lss	Lanosterol synthase (2,3-oxidosqualene-lanosterol cyclase)	620955	81681	1.41E-08	20	12533754	12534464
Med6	Mediator complex subunit 6	1310296	299180	1.09E-19	6	105446090	105446990
Mlf2	Myeloid leukemia factor 2	1306294	312709	2.46E-11	4	161059133	161059932
Mt2A	Metallothionein 2A	1592345	689415	2.25E-14	19	11268060	11268760
Mta1	Metastasis associated 1	621018	64520	1.94E-15	6	137660231	137661023
Nanos3	nanos homolog 3 (Drosophila)	1306672	288909	7.42E-08	19	25668935	25669840
Npc2	Niemann-Pick disease, type C2	628756	286898	1.69E-13	6	108814526	108815606
NSCAN Chr7.521.a	Predicted gene	NA	NA	3.28E-16	7	54504179	54505054
NSCAN ChrX.046.a	Predicted gene	NA	NA	2.35E-14	X	7961753	7962558
Olr1584	Olfactory receptor 1584	1334087	289247	9.56E-15	13	89782014	89782792
Olr40	Olfactory receptor 40	1334204	293197	3.72E-11	1	160090933	160092343
Olr425	Olfactory receptor 425	1333333	296687	3.09E-16	3	16610649	16611459
Pcdha13	Pcdha13 protocadherin alpha 13	620751	116742	3.56E-12	18	29747775	29748375
Pcyox1	Prenylcysteine oxidase 1	628652	246302	2.29E-15	4	120579932	120580828
Peli1	Pellino 1	1311199	305549	1.26E-12	14	101869844	101870444
Phox2a	Paired-like homeobox 2a	621323	116648	7.54E-10	1	159271967	159272872
Pole3	Polymerase (DNA directed), epsilon 3 (p17 subunit)	1359475	298098	2.77E-15	5	79520269	79520987
Pou4f2	POU class 4 homeobox 2	620075	171355	8.43E-31	19	31389541	31390235
Prdx5	Peroxiredoxin 5	71007	113898	1.25E-18	1	209585932	209587138

Prickle3	Prickle homolog 3 (Drosophila)	1359685	317380	1.95E-11	X	26890034	26890634
R3hdm2	R3H domain containing 2	1310066	362894	1.89E-09	7	67361803	67362403
Rbm25	RNA binding motif protein 25	1308755	366693	2.05E-09	6	107586368	107587434
RGD1305537	Similar to RIKEN cDNA 3110001I22	1305537	363528	1.60E-10	10	1787843	1788626
RGD1562673	Similar to Prostatic spermine-binding protein precursor (SBP)	1562673	363551	3.63E-08	10	13203365	13203965
Rnasek	Rnasek ribonuclease, RNase K	1305687	287453	2.15E-23	10	57077341	57078238
Rnf5	Ring finger protein 5	1588458	407784	5.15E-11	20	4247105	4248855
Rpl30	Ribosomal protein L30	621201	64640	5.15E-13	7	69889253	69890539
Rpl38	Ribosomal protein L38	1305573	689284	2.65E-51	10	104544737	104546121
Rpl41	Ribosomal protein L41	621210	124440	3.96E-20	7	1839514	1840666
RT1-M6-2	RT1 class I, locus M6, gene 2	1595815	365527	4.58E-12	20	1675848	1676448
Scyl1	SCY1-like 1 (S. cerevisiae)	1307330	293684	8.71E-08	1	208359148	208359748
Sfrp2	Secreted frizzled-related protein 2	735163	310552	1.36E-15	2	175477883	175479265
Slc10a4	Solute carrier family 10 (sodium/bile acid cotransporter family), member 4	1309536	305309	1.81E-17	14	37607465	37608065
Slc15a3	Solute carrier family 15, member 3	628663	246239	1.24E-10	1	213340695	213341500
Slc1a1	Solute carrier family 1 (neuronal/epithelial high affinity glutamate transporter, system Xag), member 1	3696	25550	8.08E-23	1	232487078	232487865
Slc35a2	Solute carrier family 35 (UDP-galactose transporter), member A2	2293497	100158233	2.17E-09	X	26650501	26651200
Slc5a5	Solute carrier family 5 (sodium iodide symporter), member 5	69267	114613	9.85E-09	16	19042786	19044400
Sms	Spermine synthase	1564826	363469	8.72E-11	X	58816696	58817492
Spata2	Spata2 spermatogenesis associated 2	620754	114210	1.62E-17	3	158640784	158641479
Spr	Sepiapterin reductase (7,8-dihydrobiopterin:NADP+ oxidoreductase)	3753	29270	1.15E-23	4	119372067	119372906
Stt3a	STT3, subunit of the oligosaccharyltransferase complex, homolog A (S. cerevisiae)	1565793	500972	4.40E-11	8	38016244	38017123
Supt16h	Suppressor of Ty 16 homolog (S. cerevisiae)	1310032	305851	8.87E-11	15	27642060	27643789
Tmem119	Transmembrane protein 119	1307573	304581	3.82E-18	12	43859955	43860555
Tmem72	Transmembrane protein 72	1308850	362424	3.82E-09	4	153065628	153066228
Tomm40b	Translocase of outer mitochondrial membrane 40 homolog B (yeast)	1562006	304971	1.07E-20	13	87111902	87112902
Tpra1	Transmembrane protein, adipocyte asscociated 1	620858	85494	5.37E-26	4	122999670	123001095
Trpv4	Transient receptor potential cation channel, subfamily V, member 4	69337	66026	1.20E-22	12	43224793	43225577
Tsku	Tsukushi small leucine rich proteoglycan homolog (Xenopus laevis)	1359311	308843	5.35E-30	1	155621758	155622558
Tyro3	TYRO3 protein tyrosine kinase	3923	25232	6.28E-11	3	106309762	106310557
Ubb	Ubiquitin B	621562	192255	1.33E-21	10	48738155	48738755
Vamp1	Vesicle-associated membrane protein 1	3948	25624	1.57E-17	4	161331679	161332357
Vcl	Vinculin	1311217	305679	1.04E-24	15	3575689	3576789
Vwa1	Von Willebrand factor A domain containing 1	1311476	298683	6.24E-14	5	172632883	172633678
Wdr13	WD repeat domain 13	1560982	317370	1.89E-10	X	26393965	26394646
Zbtb16	Zinc finger and BTB domain containing 16	727921	353227	2.32E-07	8	52095606	52096206

Regions showing vinclozolin-induced transgenerational change with MeDIP-Chip analysis of F3 generation Sertoli cells
