# Supplementary material for: Environmentally Induced Epigenetic Transgenerational Inheritance of Altered Sertoli Cell Transcriptome and Epigenome: Molecular Etiology of Male Infertility
Source: PLoS One. 2013 Mar 28;8(3):e59922. doi: 10.1371/journal.pone.0059922 (PMC3610698; doi:10.1371/journal.pone.0059922)
Supplement: Table S3 — Relations between vinclozolin-induced transgenerational DMR and expression changes in Sertoli cells. (PDF) [file pone.0059922.s006.pdf]

Supplementary Table S3.

PROPOSED INTERACTION	DMR CHANGED	DMR LOCATION REGARDING EXPRESSION CHANGES	DIFFERENTIALLY EXPRESSED GENES RELATED TO DMR
DIRECT REGULATION	Prdx5	is same gene as	Prdx5
	Pole3	is same gene as	Pole3
Total regions	*2 DMR*		*2 regions (in 2 genes) changed in gene expression*
CLUSTER REGULATION	Scyl1	is inside cluster	Tmem134, Brms1, Gene id Chr1_3166.1 (possibly Rnaseh2c), mRNA BC090353 (possibly Malat1), Znhit2, Prdx5
	Prdx5	is inside cluster	
	RGD1562673	is inside cluster	Srrm2, Pgp, Hagh
	Ubb	is within 2MB from cluster	Lrrc48, Mapk7, B9d1
	Kctd11	is inside cluster	Ccdc42, Odf4, RGD1563106, Tmem107, Dnah2, Eif4a1, Eif5a, Ybx2, Med11, Psmb6, Rpain
	Rnasek	is inside cluster	
	Nanos3	is inside cluster	RGD1564093, Junb, Syce2, Asf1b
	Gene id Chr19_655.1 (possibly Wrd59)	is inside cluster	EST CR464641 (possibly Hydin), Gene id Chr19_634.1 (possibly Hydin - region 1), Gene id Chr19_634.1 (possibly Hydin -region 2), Gene id Chr19_634.1 (possibly Hydin -region 3)
	Pole3	is inside cluster	Fkbp15, Wdr31, Bspry, Atp6v1g1
	Ccdc17	is inside cluster	
	Atp6v0b	is within 2MB from cluster	Rad54l, Faah, Pik3r3
	Med6	is inside cluster	Cwc22 (region1), Cwc22 (region 2), Cwc22 (region 3)
	Rbm25	is within 2MB from cluster	
	Ilvbl	is within 2MB from cluster	Zfp347, LOC100125368, Mrpl54, Lsm7, Atp8b3, Mum1
	Gene id Chr7_1541.1	is within 2MB from cluster	Pla2g6, Cby1, Gtpbp1, Rbx1
Total regions	*15 DMR*		*51 regions (in 46genes) changed in gene expression*
LONG DISTANCE NON-CLUSTER REGULATION	Gng8	is less than 2Mb distant from	Calm3
	Ceacam6	is less than 2Mb distant from	Exosc5
	Dll3	is less than 2Mb distant from	LOC687295
	Gene id Chr1_1386.1	is less than 2Mb distant from	EST CV117152 (possibly Il4i1)
	Tsku	is less than 2Mb distant from	Odz4
	Cox8b	is less than 2Mb distant from	Lrrc56, Pnpla2
	Slc15a3	is less than 2Mb distant from	Patl1
	Slc1a1	is less than 2Mb distant from	Jak2, Insl6
	Slc10a4	is less than 2Mb distant from	Sgcb, Tec
	Peli1	is less than 2Mb distant from	Ugp2
	Gad2	is less than 2Mb distant from	Thnsl1, Acbd5
	Mt2A	is less than 2Mb distant from	Slc38a7, Bbs2
	Pou4f2	is less than 2Mb distant from	Abce1
	Esrp2	is less than 2Mb distant from	Exoc3l
	Lhx8	is less than 2Mb distant from	Usp33
	Bat2l	is less than 2Mb distant from	Pomt1
	Tyro3	is less than 2Mb distant from	Rpusd2, Casc5
	Hdc	is less than 2Mb distant from	Fbn1, Gene id chr3_1591.1 (possibly Ncaph), Gpat2
	Acoxl	is less than 2Mb distant from	Gene id chr3_1591.1 (possibly Ncaph), Gpat2
	Atp6v0b	is less than 2Mb distant from	Ppih
	Emilin1	is less than 2Mb distant from	Hadhb
	Rbm25	is less than 2Mb distant from	Sipa1l1, Eif2b2
	Npc2	is less than 2Mb distant from	Eif2b2
	Rpl41	is less than 2Mb distant from	Sarnp
	NSCAN Chr7.521.a	is less than 2Mb distant from	LOC362855
	Rpl30	is less than 2Mb distant from	RGD1565493 (region 1), RGD1565493 (region 2)
	Gene id Chr7_1541.1	is less than 2Mb distant from	Pole3
	Krt83	is less than 2Mb distant from	EST FQ115996 (possibly Larp4)
	Kb15	is less than 2Mb distant from	EST FQ115996 (possibly Larp4), Gtsf1
	Stt3a	is less than 2Mb distant from	Ei24
	Zbtb16	is less than 2Mb distant from	Pih1d2
Total regions	*31 DMR*		*39 regions (in 38 genes) changed in gene expresion*

Relations between vinclozolin-induced transgenerational DMR and expression changes in Sertoli cells
