# Supplementary material for: Environmentally Induced Epigenetic Transgenerational Inheritance of Altered Sertoli Cell Transcriptome and Epigenome: Molecular Etiology of Male Infertility
Source: PLoS One. 2013 Mar 28;8(3):e59922. doi: 10.1371/journal.pone.0059922 (PMC3610698; doi:10.1371/journal.pone.0059922)
Supplement: Table S4 — Clusters of transgenerational changes in gene expression and their relation to DMR in vinclozolin-lineage F3 Sertoli cells. (PDF) [file pone.0059922.s007.pdf]

Supplementary Table S4

	CLUSTER LOCATION	DMR INSIDE OR WITHIN 2MB OF A CLUSTER	GENES WITH EXPRESSION CHANGE IN THE CLUSTER
1	chr1:39150000:42350000		Ldhal6b, Tmem181, Dynlt1
2	chr1:62450000:65850000		Eif5b (chr1), Cnot3, E2f5, Aurkc
3	chr1:206250000:210350000	Scyl1, Prdx5 (inside cluster)	Tmem134, Brms1, Gene id Chr1_3166.1 (possibly Rnaseh2c), mRNA BC090353 (possibly Malat1), Znhit2, Prdx5
4	chr10:12150000:15050000	RGD1562673 (inside cluster)	Srrm2, Pgp, Hagh
5	chr10:45700000:48600000	Ubb (within 2MB from cluster)	Lrrc48, Mapk7, B9d1
6	chr10:53800000:59250000	Kctd11, Rnasek (inside cluster)	Ccdc42, Odf4, RGD1563106, Tmem107, Dnah2, Eif4a1, Eif5a, Ybx2, Med11, Psmb6, Rpain
7	chr10:88500000:92250000		Tubg1, Ccdc56, Rpl27, LOC688211
8	chr12:32400000:36300000		Rsrc2, Diablo (chr12), Gene id Chr12_661.1 (possibly Bcl7a), Wdr66, Rad9b
9	chr14:8150000:11050000		Cds1, Mrps18c, Helq
10	chr14:81900000:85550000		Tacc3, Atp5e, Drg1, LOC685322
11	chr16:30450000:33850000		Cbr4, Sh3rf1, Nek1
12	chr17:48150000:52050000		RGD1564767, Zfp322a, LOC680322, Hist1h2bn, Zfp187
13	chr19:22950000:26800000	Nanos3 (inside cluster)	RGD1564093, Junb, Syce2, Asf1b
14	chr19:38500000:42400000	Gene id Chr19_655.1 (possibly Wrd59) (inside cluster)	EST CR464641 (possibly Hydin), Gene id Chr19_634.1 (possibly Hydin - region 1), Gene id Chr19_634.1 (possibly Hydin -region 2), Gene id Chr19_634.1 (possibly Hydin - region 3)
15	chr2:188900000:191150000		Zc3h11a, Psmb4, Vps45
16	chr3:3150000:7800000		Ssna1, Qsox2, Mrpl41 (chr3), Rpl7a (region 1), Rpl7a (region 2), Surf4
17	chr3:168850000:171000000		Gene id Chr3_2440.1 (possibly Lsm14b), RGD1305899, Tcea2
18	chr5:77500000:81400000	Pole3, Ccdc17 (inside cluster)	Fkbp15, Wdr31, Bspry, Atp6v1g1
19	chr5:134600000:138150000	Atp6v0b (within 2MB from cluster)	Rad54l, Faah, Pik3r3
20	chr6:102050000:105600000	Med6 (inside cluster), Rbm25 (within 2MB from cluster)	Cwc22 (region1), Cwc22 (region 2), Cwc22 (region 3)
21	chr7:7950000:12350000	Ilvbl (within 2MB from cluster)	Zfp347, LOC100125368, Mrpl54, Lsm7, Atp8b3, Mum1
22	chr7:116000000:119850000	Gene id Chr7_1541.1 (within 2MB from cluster)	Pla2g6, Cby1, Gtpbp1, Rbx1
23	chr8:73000000:75750000		Rora, Narg2, Rnf111, Adam10
24	chr9:72550000:75700000		Gene id Chr9_929.1 (possibly Usp37), Stk36, Gene id Chr9_958.1

Clusters of transgenerational changes in gene expression and their relation to DMR in vinclozolin-lineage F3 Sertoli cells
